# Development and Experimental Validation of a Dispersity Model for *In Silico* RAFT Polymerization

**DOI:** 10.1021/acs.macromol.2c01798

**Published:** 2023-02-09

**Authors:** Clarissa. Y. P. Wilding, Stephen. T. Knox, Richard. A. Bourne, Nicholas. J. Warren

**Affiliations:** † School of Chemical and Process Engineering, University of Leeds, LS2 9JT Leeds, U.K.; ‡ Institute of Process Research and Development, School of Chemistry, University of Leeds, LS2 9JT Leeds, U.K.

## Abstract

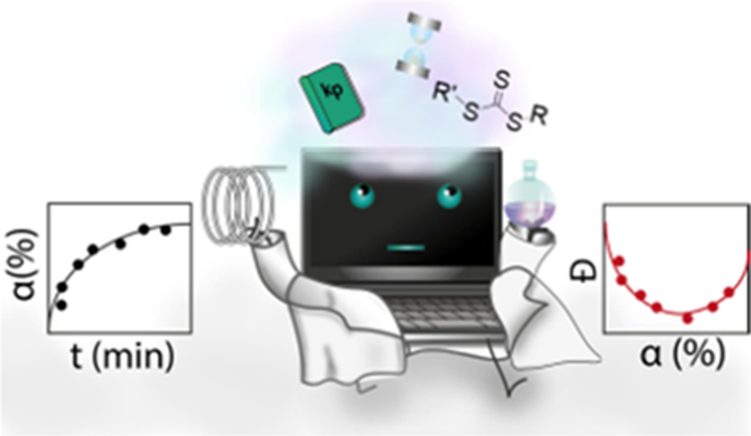

The exploitation of computational techniques to predict the outcome of chemical reactions is becoming commonplace, enabling a reduction in the number of physical experiments required to optimize a reaction. Here, we adapt and combine models for polymerization kinetics and molar mass dispersity as a function of conversion for reversible addition fragmentation chain transfer (RAFT) solution polymerization, including the introduction of a novel expression accounting for termination. A flow reactor operating under isothermal conditions was used to experimentally validate the models for the RAFT polymerization of dimethyl acrylamide with an additional term to accommodate the effect of residence time distribution. Further validation is conducted in a batch reactor, where a previously recorded *in situ* temperature monitoring provides the ability to model the system under more representative batch conditions, accounting for slow heat transfer and the observed exotherm. The model also shows agreement with several literature examples of the RAFT polymerization of acrylamide and acrylate monomers in batch reactors. In principle, the model not only provides a tool for polymer chemists to estimate ideal conditions for a polymerization, but it can also automatically define the initial parameter space for exploration by computationally controlled reactor platforms provided a reliable estimation of rate constants is available. The model is compiled into an easily accessible application to enable simulation of RAFT polymerization of several monomers.

## Introduction

Reversible deactivation radical polymerization (RDRP) techniques have revolutionized polymer synthesis since their conception in the late 20th century.^1−4^ They enable the synthesis of well-defined vinyl (co)polymers with targeted molecular weight and low molar mass dispersity (*Đ*) without the need for stringent synthetic procedures associated with techniques such as living anionic polymerization. The three most studied RDRP techniques, atom transfer radical polymerization (ATRP),^1,5,6^ nitroxide mediated polymerization (NMP),^7−10^ and reversible addition fragmentation chain transfer (RAFT),^2,11−13^ all have well-studied and well-understood mechanisms,^14,15^ with pseudo-first-order kinetics, a linear evolution in number-average molecular weight (*M*_n_) with conversion (α), and resulting low-*Đ* polymers (typically < 1.20). These properties are a result of the equilibrium between the dormant species and propagating radicals; in the absence of this (for FRP), broader statistical distributions of molecular weights are observed.^16^

In the context of RDRP, mathematical models are shown to be useful in predicting outcomes such as conversion, molecular weight distributions (MWD), and dispersity, but most require a deep understanding of the mechanisms and rate constants. Both deterministic and stochastic approaches have been employed to model ATRP,^17,18^ NMP,^19,20^ and RAFT,^21−23^ where deterministic techniques require the solution of ordinary differential equations (ODEs) and differential algebraic equations (DAEs), while stochastic techniques involve the probabilities of success of discrete reaction events. Although stochastic methods such as Monte Carlo (MC) simulation allow more information about topological architecture and intramolecular interactions to be obtained, they are much more computationally expensive than deterministic techniques.^20,24,25^

Typically, for RAFT and other RDRP techniques, experimental kinetics are obtained by monitoring changes in conversion and molecular weight with respect to time. Monitoring *M*_n_ and dispersity can enable mechanistic insights and indicate the presence of side reactions. Both deterministic and stochastic approaches can be used to model the kinetic profiles of RAFT with temporal resolution.^21,22^ Commonly, simultaneous numerical methods are used to solve the series of rate equations; however, an example of algebraic-type simplification of the rate ODEs to quantify monomer conversion has been demonstrated.^18^ Termination and transfer events make a significant contribution to the statistical distribution of molecular weights—increased termination at higher conversions is shown to cause a broadening in MWDs, leading to an upturn in *Đ* as the reaction progresses. Literature simulations focus on the significant retardation of the overall rate of polymerization caused by the addition of dithiobenzoate (DTB) compounds compared to FRP. It has been shown that varying levels of retardation occur in trithiocarbonates (TTC) and xanthates.^23^ The mechanism of rate retardation is debated by polymer chemists, with three main theories:^23^ intermediate radical termination,^24^ slow fragmentation method,^19^ and missing step reaction.^26−30^

Commonly, the commercially available modular deterministic software PREDICI (which utilizes a discretized Galerkin *h-p* method) can be applied to most polymerizations and provides a flexible method of predicting conversion and full MWDs. PREDICI allows microstructural and topological information to be obtained by accounting for arbitrary numbers of species, distributions, reaction steps, and avoiding mechanistic assumptions (e.g., steady-state hypothesis).^31−35^ PREDICI has been applied and experimentally validated several times in the literature for RAFT.^36^ In the last decade, PREDICI has enabled the determination of rate coefficients for unusual monomers,^37^ simulation of chain extensions and the effect on “livingness”,^38^ and for optimization of reactor vessels.^33^ However, it is not open access and requires the user to know the mechanistic pathways. Alternatively, method of moments has become a popular deterministic approach due to its low computational cost—where discretization of each kinetic step enables simplification.^39^ Deterministic techniques can be made computationally less expensive through the pseudo-steady-state approximations (PSSA), which decreases the stiffness of the ODEs and DAEs. Full elucidation of the chain-length distribution has been reported in the literature using PSSA deterministic techniques, using direct integration of the living radical species, even for mechanisms where rate retardation is governed by IRT or SFM.^40^ Finally, a modified Monte Carlo (MC) simulation of RAFT polymerization has been demonstrated at reduced computational expense using different programming languages with Julia computing MWDs in less time than MATLAB, Python, and FORTRAN.^41^

Explicit quantitative models for dispersity are attractive due to their ease of use, open accessibility, no need for high-performance PCs, and the ability to code into a range of software packages. Zhu and co-workers derived dispersity as a composite equation for RDRP comprising a living step, transfer steps, and terminative steps.^42,43^ Currently, only full equations for normal ATRP^42^ and NMP^44^ have been derived (Table 1) by employing blend and block theory. For ATRP and NMP, activation/deactivation effects dominate during the initial stages of the polymerization, where chains are relatively short, but it is commonly speculated that terminative events become more significant during the later stages, where the polymer chains are much longer.^42,43,45^ Work simulating the molecular weight distributions for ATRP, RAFT, and cationic polymerizations based on the first three terms of the dispersity equation that exist in the literature have been fitted to experimental data to provide information about the control.^46^ Terminative events are quantified in the final term of both equations for ATRP and NMP and manifest in an increase in *Đ*, but we are not aware of an equivalent term for RAFT polymerization.^47^

**Table 1:** Existing Models for Mass Dispersity for ATRP and NMP and for RAFT Polymerization^a^

	quantitative equation
ATRP^42^	
NMP^44^	
RAFT (this work)	

a Here, *k*_act_, *k*_deact_, *k*_p_, *k_t_*, *k*_tr_, and *k*_–tr_ are the rate constants for activation, deactivation (ATRP and NMP), propagation, termination, forward transfer, and backward transfer (RAFT only), respectively. Initial concentrations of the radical generating species ([*P_n_X*]_0_), monomer ([*M*]_0_), and catalyst species ([*C*]_0_ and [*XC*]_0_) and RAFT agent concentration [*CTA*]_0_. Conversion is denoted as α.

Herein, we couple a modified kinetic model based on ODEs with a model for molar mass dispersity (Table 1), which includes a novel term accounting for terminative events during the later stages of RAFT polymerization. This enables more accurate prediction of conversion and dispersity with an opportunity of narrowing initial parameter space for computationally directed polymer discovery.

## Results and Discussion

### Kinetic Model

To model the consumption of the monomer, a series of ODEs are constructed to describe the kinetic parameters for the reaction (Table 2) and solved for conversion. The Arrhenius equation is used to account for temperature in the rate constants. The concentration of chain species, propagating radicals (*P_r_*), chain transfer agent species (*CTA*), radical adduct intermediates (*CTA_a_*), linear polymer chains (*P*), and branched chains (*P*′), are assumed to be independent of the chain length. [*CTA*] described in (iv) is a summation of all chain transfer species including the initial [*CTA*] at time = 0. *R* seen in the pre-equilibrium represents the leaving group of the RAFT agent, while *P*_*r*_ represents any length of propagating chain. It is important to note that *r* in *P*_*r*_ does not indicate the length of the macroradical. Steady-state hypothesis is applied to enable the simplification of the equations to an ODE for  and then solved using the *symbolic math toolbox* in MATLAB. This was then used to find [*P*_*r*_] at steady state enabling solution of (ii) for monomer concentration, [*M*]_*t*_ at a given time and thus conversion (eq 1).
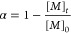
1Once [*M*]_*t*_ is determined for non-chain-length-dependent reaction, a second iteration is performed accounting for chain-length-dependent termination (CLD-T).^48^ This involves a cross-over chain length where the termination rate operates using two separate equations for calculating *k*_*t*_: short chain (*L* < *L*_c_) and long chain (*L* > *L*_c_). This cross-over chain length is typically identified experimentally by single-pulse pulsed-laser polymerization (SPPLP) coupled to electron paramagnetic resonance spectroscopy (EPR).^49^ A log plot of the radical concentration, *c*_R_, at *t* = 0 and after the pulse vs time enables the fitting and subsequent *L*_c_ and power laws to be obtained (see the Supporting Information).^50^

**Table 2:** Steps Describing the General RAFT Mechanism and Rate Equations for Each Species

step		rate equation	#
initiation			i
propagation			ii
pre-equilibrium			iii
reinitiation			iv
main equilibrium			v
termination by disproportionation			vi
termination by coupling			vii
cross-termination			

The inaccessibility of rate constants in the literature is often stated as the primary issue when modeling RAFT;^51^ thus, it is important to note the dependence of the model on explicit rate constants. The model relies on five rate constants: *k*_p_, *k*_d_, *k_t_*, *k*_a_, and *k*_β_, where *k*_p_ and *k_t_* are the most studied experimentally using pulsed-laser polymerization (PLP) combined with SEC and electron spin resonance spectroscopy (ESR).^52−54^*k*_d_ values are also abundant in the literature and are typically found by measuring gas evolution with respect to time.^55,56^ Less commonly studied are *k*_a_, *k*_–a_, *k*_β_, and *k*_–β_ which are uniquely associated with RAFT polymerization. *k*_a_ is typically calculated from the chain transfer coefficient obtained experimentally by a Mayo plot or by comparing monomer conversion to RAFT agent conversion.^57^ Efforts to quantify *k*_β_ via the RAFT equilibrium constant have been limited to polymerizations exhibiting rate retardation. This is carried out by comparing rates of polymerization at different concentrations of RAFT agent^58^ and through *ab initio* studies.^59^

**Figure 1:**
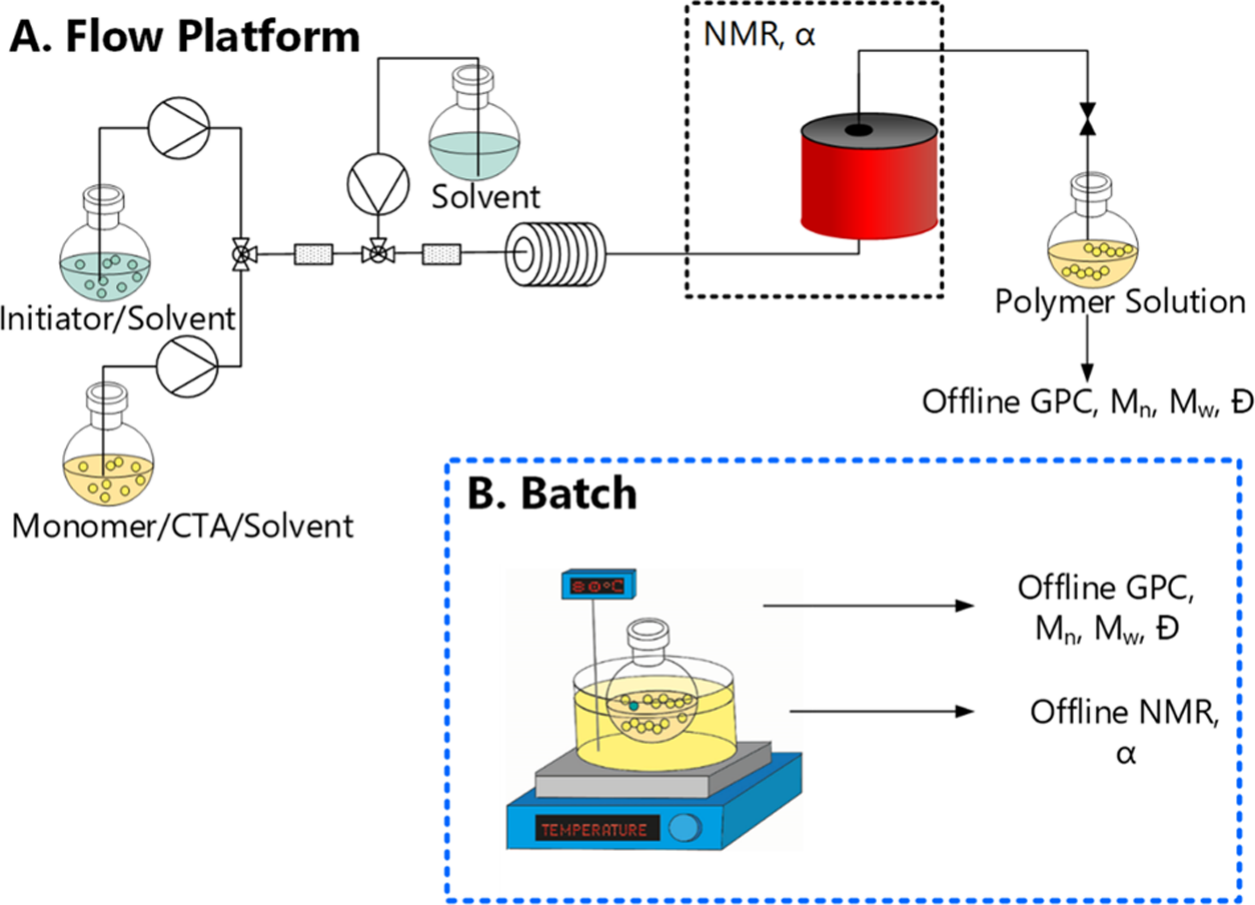
Schematics of the (A) flow reactor platform, consisting of a heated 5 mL coil coupled with inline GPC and offline NMR, and (B) batch reactor. Offline analysis is performed for both methods.

**Figure 2:**
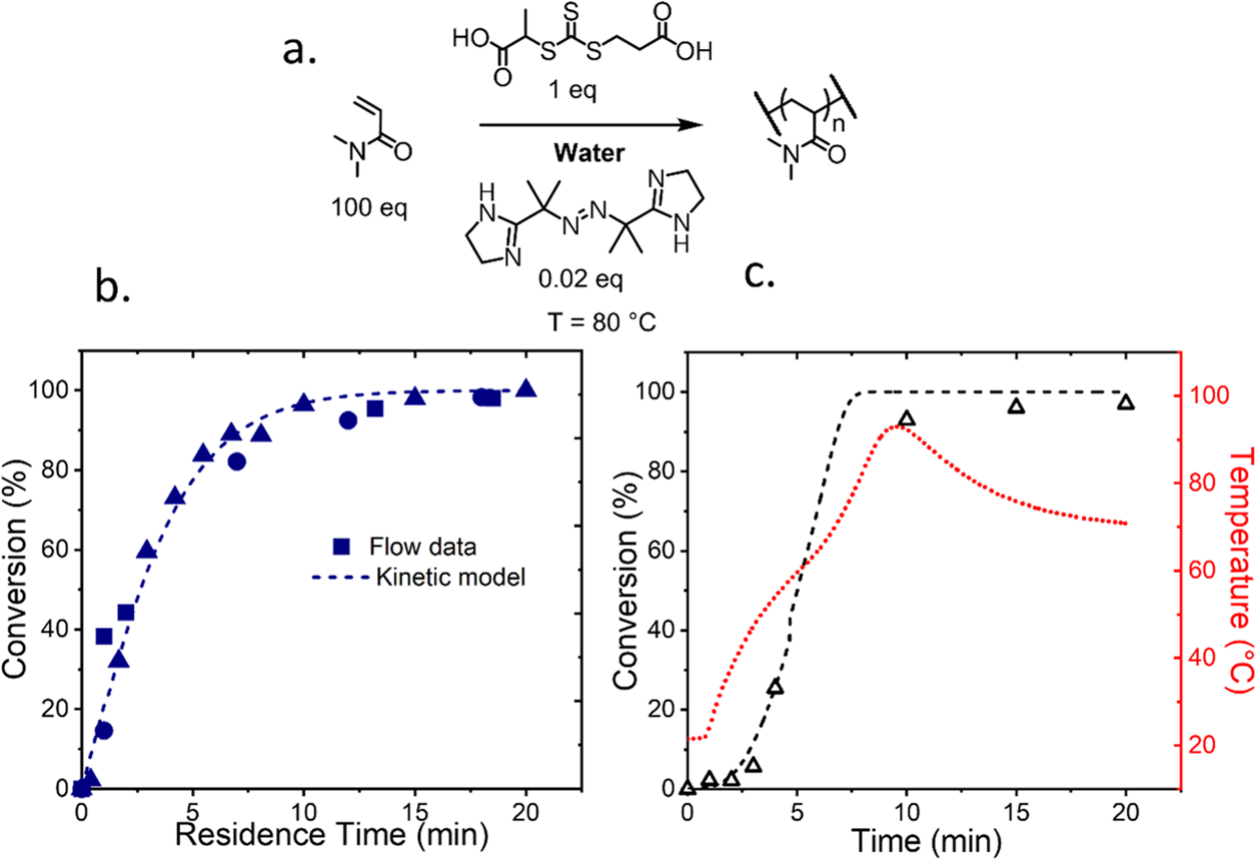
(a) Reaction scheme for the aqueous solution ultrafast RAFT polymerization of DMAm in the presence of TTC1 using VA044 as the initiator, in a 100:1:0.02 ratio, respectively, at 30 w/w % at 80 °C. (b) Simulated kinetics (dashed line) are compared to experimental results for the flow reactor (data points) where squares, circles, and triangles represent separate runs of the same reaction (c) In batch, the nonisothermal kinetics (black) are simulated using the temperature measured *in situ* (red line). The temperature profile illustrates the poor heat transfer leading to an initial induction period and subsequent polymerization exotherm.

An initial simulation was performed for the RAFT polymerization of dimethyl acrylamide (DMAm) under ideal “isothermal” conditions in water and compared to data obtained experimentally in batch and flow (Figures 1 and 2). This system was chosen as it is widely studied^60,61^ and the propagation constants are widely available.^62,63^

To best reproduce isothermal conditions (Figure 2b), the polymerization was conducted in a flow reactor, where the higher surface area-to-volume ratio facilitated superior heat transfer. In this case, the experimental data were in good agreement with the model (dashed line), exhibiting the expected pseudo-first-order kinetics.

An equivalent batch reaction was also conducted, whereby the ambient temperature reaction solution was immersed in an oil bath at 80 °C. Experimental data indicated a delayed onset of polymerization followed by a large increase in conversion over a short time interval. This did not align with the isothermal kinetic model due to the poor heat transfer. An initially slow polymerization was observed, which auto-accelerated due to poor dissipation of the exotherm, as seen in the peak in temperature peak above 90 °C, which can be seen from *in situ* temperature monitoring (Figure 2c). From this temperature profile, a semiempirical model was built, which considers the varying temperature which overlays well with the experimental data, demonstrating the wide applicability of this kinetic model. Subsequently, the temperature dependence was then investigated in flow for a different RAFT agent and initiator combination using a higher monomer concentration to ensure a dynamic model for simulating ideal systems.

**Figure 3:**
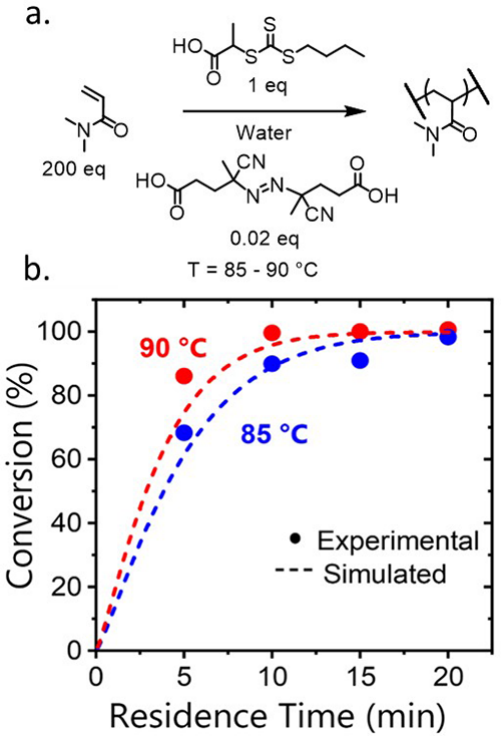
(a) Reaction scheme for the aqueous solution RAFT polymerization of DMAm in the presence of TTC2 using ACVA as the initiator, in a 200:1:0.02 ratio, respectively, at 30 w/w %. (b) Comparison of kinetic conversion data obtained in flow (filled circles) at different temperatures. Here, the color of the symbol and dashed line correspond to different temperatures, 85 °C (blue) and 90 °C (red), and simulation at the corresponding temperature.

The simulated conversion traces again show good concordance with the experimental flow data even when an initiator with a slower rate of decomposition is used (Figure 3). It is increasingly important to consider the temperature dependence for radical polymerizations, as highlighted in Figure 3 by the increase in the rate of reaction observed when the temperature is elevated by 5 °C. This expected increase confirms that assuming Arrhenius behavior is satisfactory for this reaction system. For bulky acrylate polymerizations where high temperature can lead to increased rate of side reactions (e.g., formation of mid-chain radicals), a reduced polymerization rate can be observed—in which case the model would become invalid.

#### Derivation of Dispersity Equation

For “living” polymerization (no terminative or reversible transfer steps), the dispersity decreases asymptotically as a function of conversion (eq 2, where  and MWD is typically a Poisson distribution).^64^ Following block theory, which assumes that there is no termination after each time step,^65^eq 2 has been defined for completely living polymerization.
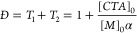
2where [*CTA*]_0_ and [*M*]_0_ are the initial concentrations of *CTA* and monomer, respectively. For simplicity, here, we abbreviate each term derived as *T*_*n*_, where *T*_1_ is the first term, *T*_2_ is the second term, etc. Due to the reversible activation/transfer steps involved in RDRP, the term previously derived by Harrisson *et al*.^47,64^ can be added, resulting in an equation for dispersity as a function of conversion
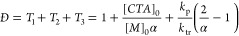
3where [*CTA*]_*t*_ is the concentration of *CTA* at time, *t*, and *k*_p_ and *k*_tr_ are the rate constants for propagation of radicals and transfer of monomer to *CTA*, respectively. This step broadens the MWD leading to slightly higher *Đ*. Harrisson *et al*.^64^ further simplify the formula by assuming that the ratio of  = 1 for the ideal case.

To provide a further improvement in the prediction of dispersity, a fourth term, *T*_4_, is necessary to account for terminative events that lead to dead polymer chains.

**Figure 4:**
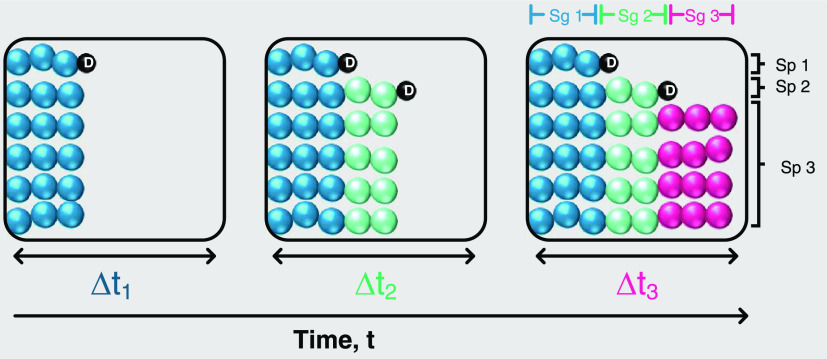
Schematic of how the model describes chain growth in CRP based on the blend and block strategy demonstrated by Mastan *et al*.^42^ Sg # = Segment and Sp # = Subpopulation. The black spheres labeled “*D*” represent dead polymer in the reaction. The model assumes that after each time step, Δ*t*_*i*_, there is a degree of livingness and termination such that, in Δ*t*_1_, Sg 1 terminates to form Sp 1 but Sg 2 grows, and in Δ*t*_2_, Sg 2 terminates forming Sp 2 and Sg 3 grows, etc.

Here, blend and block theory (Figure 4) was used as the basis to achieve an explicit value for *T*_4_—chain growth and terminative subpopulations are discretized per time interval to quantify *Đ* as a function of time and, in turn, conversion.

The model assumes that a thermally initiated polymerization will begin instantaneously on introduction of radicals, i.e., as soon as the reaction medium is heated. A further major assumption is that radical concentration is at steady state in each time interval; thus, if all of the initiator radicals have been consumed (i.e., at high temperatures at long reaction times), then the model will become invalid. Realistically, all radicals may be consumed under intense conditions, leading to rate retardation and reduced conversion as the concentration of dead polymer increases.

**Figure 5:**
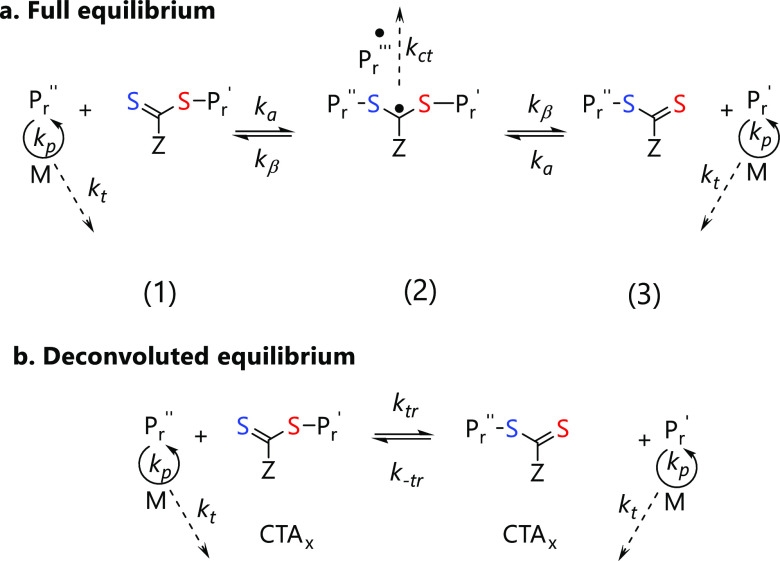
(a) Complete RAFT equilibrium following, highlighting the mechanism of chain transfer. Addition (*k*_a_) of *P*_*r*_ to *CTA* (1), then β scission (*k*_β_) of radical adduct intermediate (2) to form *CTA* (3). Intermediate (2) can also undergo cross-termination (*k*_ct_) to form branched polymer species. In RAFT, termination (*k_t_*) and propagation (*k*_p_) are also happening at the same time. (b) Simplification of RAFT equilibrium where *k*_tr_ and *k*_–tr_ account for *k*_a_, and *k*_β_ and the partitioning of species (2).^66,67^

To build an effective model, it is critical to understand how the RAFT equilibrium (Figure 5) impacts the dispersity. *CTA* design is important in polymerization control, whereby the stability of the intermediate and slow rate of addition/fragmentation can cause retardation. Additionally, compatibility of *CTA* with the monomer is equally important and is dictated by the activity of the Z and R group.^68^ The model derived here is based on the well-controlled and widely used polymerization of activated monomers (MAMs) in the presence of trithiocarbonate (TTC)-based *CTA*s. Cross-termination is neglected due to the instability of the radical adduct species (*k*_ct_ = 0), and the full equilibrium (Figure 5a) can be simplified by accounting for partitioning of the radical adduct intermediate between starting materials and products (Figure 5b).^66,67^ The ratio of transfer to propagation can then be described as *C*_tr_ = *k*_tr_/*k*_p_, which is known as the chain transfer coefficient. The transfer rate constant *k*_tr_ accounts for addition, fragmentation, and the partitioning of the radical adduct species between reactants and products in the RAFT equilibria. To obtain good control, associated with low *Đ* (*Đ* < 1.3) polymers, a higher *k*_tr_ is preferred, which increases the value of *C*_tr_.^69−71^ Blend and block theory^42^ used in this paper assumes that the degree of polymerization of each segment is the product of the number of monomeric units added per transfer step in the equilibria and that the total DP will be a sum of the DP of each polymer chain after each Δ*t*_*i*_. The number of monomers added per cycle is given by looking at the probability of propagation with respect to other reactions that occur in the forward equilibria (Figure 5b) and the number of transfer steps is backward transfer step per Δ*t*_*i*_. The total DP can then be solved as the sum of all segments, which can be integrated by taking the limit as Δ*t*_*i*_ as it approaches zero.

The mass dispersity of the polymer will be a sum of the dispersity after each time interval, again taking the limit as Δ*t*_*i*_ as it approaches zero, Δ*t*_*i*_ → 0. For RAFT, there will be a fraction of terminating chains forming subpopulations and a fraction of living chains that can continue growing. The termination fraction is given by the ratio of polymer to *CTA* concentration and can be seen in *T*_4_ in eq 4. Based on the assumptions above, the following equation for RAFT is obtained:
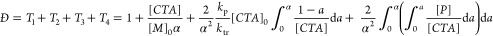
4Given that the concentration of polymer is found by integrating the rate of formation of polymer chains over time, we can then substitute, [*P*] = *k*_*t*_ [*P*_*r*_]^2^*t*, where time *t* is an unwanted variable that can instead be expressed as a function of conversion () such that
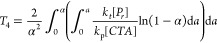
5A value of *T*_4_ can be obtained using initial concentrations and rate constants (*k*_p_, *k_t_*, *k*_tr_, *k*_–tr_, and *k*_d_). As [*P*_*r*_] is dictated by initiation rate and the ability of the *CTA* to hold propagating radicals in equilibria, this is taken into account in the model. It is also important to highlight that the value of *k*_β_ (Figure 5) is widely debated in the ITM and SFM models for certain RAFT agents.

Under the quasi-steady-state approximation, the change in concentration of propagating radicals does not change in a given time interval, so . Here, the concentrations of *CTA* species that exist for the forward and backward reactions are given by [*CTA*_*x*_] and [CTA_*y*_], respectively.
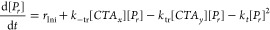
6The relationship between the concentrations of propagating radical species and initiator radicals is proportional in nature; accordingly, the rate of initiation has been accounted for in eq 6.^11^ The overall concentration of reactive radicals changes over the course of the reaction due to the decreasing concentration of initiator and the increase in terminative events. The model assumes that there will be a constant supply of radicals due to radical regeneration. By assuming degeneracy of the RAFT equilibrium such that *k*_tr_ = *k*_–tr_, the terms describing the equilibrium can be removed.

Here, it is assumed that the sum of all chain transfer species does not change over time, with very little quenching/loss of the radical adduct intermediate. It is also assumed that [*CTA*] = [*CTA*_*x*_] ≈ [*CTA*_*y*_] so the rate of transfer is dictated by the rate constants *k*_tr_ and *k*_–tr_. Consequently, we can assume that [*CTA*] at a given conversion is the same as the initial concentration ([*CTA*] = [*CTA*]_0_). If , then the quadratic eq 6 can then be solved for [*P*_*r*_], where the positive solution is obtained using the *symbolic math toolbox* in MATLAB on the basis that there cannot be negative concentration of radicals. This value quantifying the concentration of propagating radicals is substituted into eq 5, following the method of integration demonstrated in Mastan *et al*.^42^ Gaussian quadrature with one node is used to solve the double integral, which is subsequently written as a Taylor expansion with a single term. Through truncation of the infinite Taylor series, a simple formula can be obtained, but this is only an approximation and the calculation of the true value of *T*_4_ would require computational intervention. A more accurate mathematical treatment is possible, whereby the integral is solved analytically (see eq S55) and expressed as a Taylor expansion with one and two terms. This increases *Đ*, but the experimental data more closely agree with the simpler treatment. This indicates that the assumptions in the mathematical model are insufficient to account for the complexity of the polymerization system. This includes the neglecting of chain transfer to solvent, which could lead to an overestimation of *T*_4_.

**Figure 6:**
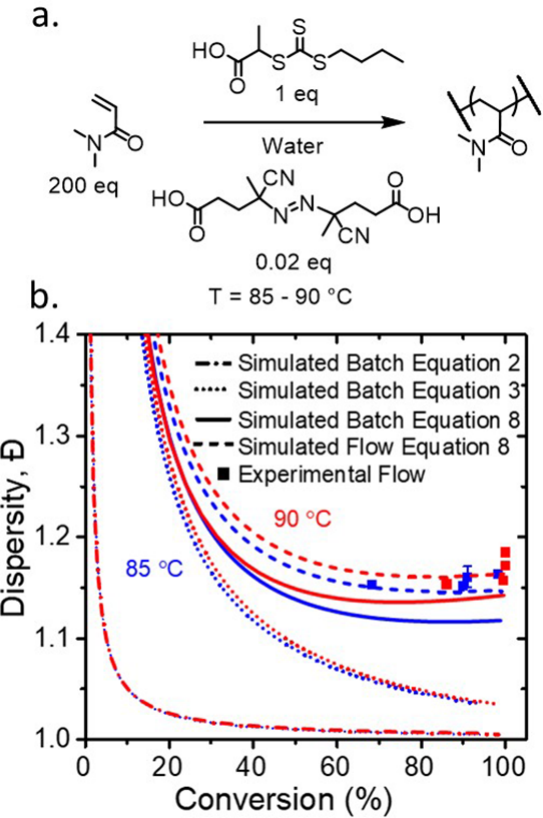
(a) Reaction scheme for the aqueous solution RAFT polymerization of DMAm in the presence of TTC2 using ACVA as the initiator, in a 200:1:0.1 ratio, respectively, at 30 w/w %. (b) Comparison of experimental dispersity and conversion (squares) obtained in flow versus the simulated batch (solid line) and flow (dashed line) reaction using eq 8. Monomer conversion is obtained via online flow-NMR, and molecular weight distributions are obtained using an offline gel permeation chromatography (GPC) calibrated with poly(methylmethacrylate) (PMMA) standards. The data shown here are subsequently corrected to consider the residence time distribution within the reactor (see Supporting Information, SI). The simulated dispersity using eqs 2 and 3 does not account for termination.

An approximate value for term 4, *T*_4_, is given by eq 7
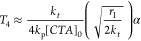
7
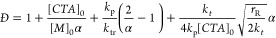
8The simulated data obtained from eq 2 exhibit the decrease in dispersity at low conversion expected for a living polymerization. In Figure 6, eq 3 accounts for chain transfer steps, causing increased dispersity, particularly at low conversions. However, solely accounting for chain growth, monomer/*CTA* transfer is insufficient at high conversion. Termination events must be considered, as in eq 8, which result in a minimum and then an upturn at intermediate conversion where the dispersity begins to gradually increase (Figure 6) similar to that seen for ATRP and NMP.^42,44^

### Validation of Dispersity Equation

Comparing the simulated data generated from eq 8 at two temperatures with the experimental data, the data at 85 °C lie on the simulated trace, suggesting that the model works well for this system. Although the use of flow chemistry has advantages in the context of efficient heat transfer, the fluid dynamics mean an inherent feature is a residence time distribution (RTD), which causes higher dispersity^72^ even in narrow tubing (1/16″). Consequently, the model needs an additional term to account for this (see the SI for incorporation of RTD on MWD). Assuming that the residence time of each polymer chain at a set flow rate can lie anywhere in the RTD, the RTD function (*E*(θ)) is superimposed on each molecular weight in the MWD forming a distribution of distributions. A fitting function is used in MATLAB to obtain the Gaussian fitting parameters. Using the fitting parameters, the Gaussians are simulated and merged. The dispersity can then be calculated and the RTD contribution determined by subtraction. It is important to note the effect of viscosity on the RTD seen in the SI, as the viscosity increases with the degree of polymerization, the dispersity will also increase.^72^

Following successful validation for DMAm, literature values for the solution RAFT polymerization of acrylamide (AAm),^73^ acrylic acid (AA), and methyl acrylate (MA)^74^ were compared to the model. First, the reported experimental conversion was entered into eq 8, then the conditions were simulated using the kinetic model coupled to eq 8. The resultant data can be seen in Table 3 (for rate parameters, see the SI). For acrylic acids, the presence of the acid group can cause issues, and so often rate parameters for *k*_p_ account for the pH.^54^

**Table 3:** Comparison of Literature Experimental Data Conducted in Batch (Conversion, α, and Dispersity, *Đ*^GPC^) to the Dispersity Obtained by Substituting the Experimental Conversion into Equation 8*Đ*^th^, and Fully Simulated Conversion, α^si^, and Dispersity, *Đ*^si^^a^

	monomer	solvent	*CTA*	initiator	[*CTA*]:[*I*]	concentration (% w/w)	*T* (°C)	*t* (min)	α (%)	*Đ*^GPC^	*Đ*^old^	*Đ*^th^	α^si^ (%)	*Đ*^si^	ref
1	acrylamide	H_2_O	TTC3	VA044	10:1	15	45	427	87	1.20	1.01	1.20	91	1.20	(73)
2	acrylamide	H_2_O	TTC3	VA044	5:1	15	45	310	97	1.20	1.01	1.26	92	1.24	(73)
3	acrylamide	H_2_O	TTC3	VA044	5:1	15	45	250	86	1.17	1.01	1.26	87	1.21	(73)
4	acrylic acid	H_2_O	TTC4	ACVA	10:1	13	69	360	97	1.18	1.01	1.17	98	1.17	(75)
5	methyl acrylate	toluene	TTC5	AIBN	10:1	30	50	199	38	1.16	1.05	1.10	16	1.15	(74)
6	methyl acrylate	toluene	TTC5	AIBN	10:1	30	50	360	51	1.15	1.04	1.10	34	1.10	(74)
7	methyl acrylate	toluene	TTC5	AIBN	10:1	30	50	399	56	1.10	1.03	1.10	39	1.10	(74)
8	methyl acrylate	toluene	TTC5	AIBN	10:1	30	50	1236	85	1.12	1.02	1.11	89	1.12	(74)
9	*N*,*N*-dimethyl acrylamide	water	TTC1	AIBN	50:1	30	80	6	67	1.15	1.07	1.12	85	1.13	
10	*N*,*N*-dimethyl acrylamide	water	TTC1	AIBN	50:1	30	80	15	94	1.17	1.04	1.13	99	1.14	
11	*N*,*N*-dimethyl acrylamide	water	TTC1	AIBN	50:1	30	80	20	97	1.19	1.04	1.13	99	1.14	

a *T* = temperature, *t* = reaction time.

**Figure 7:**
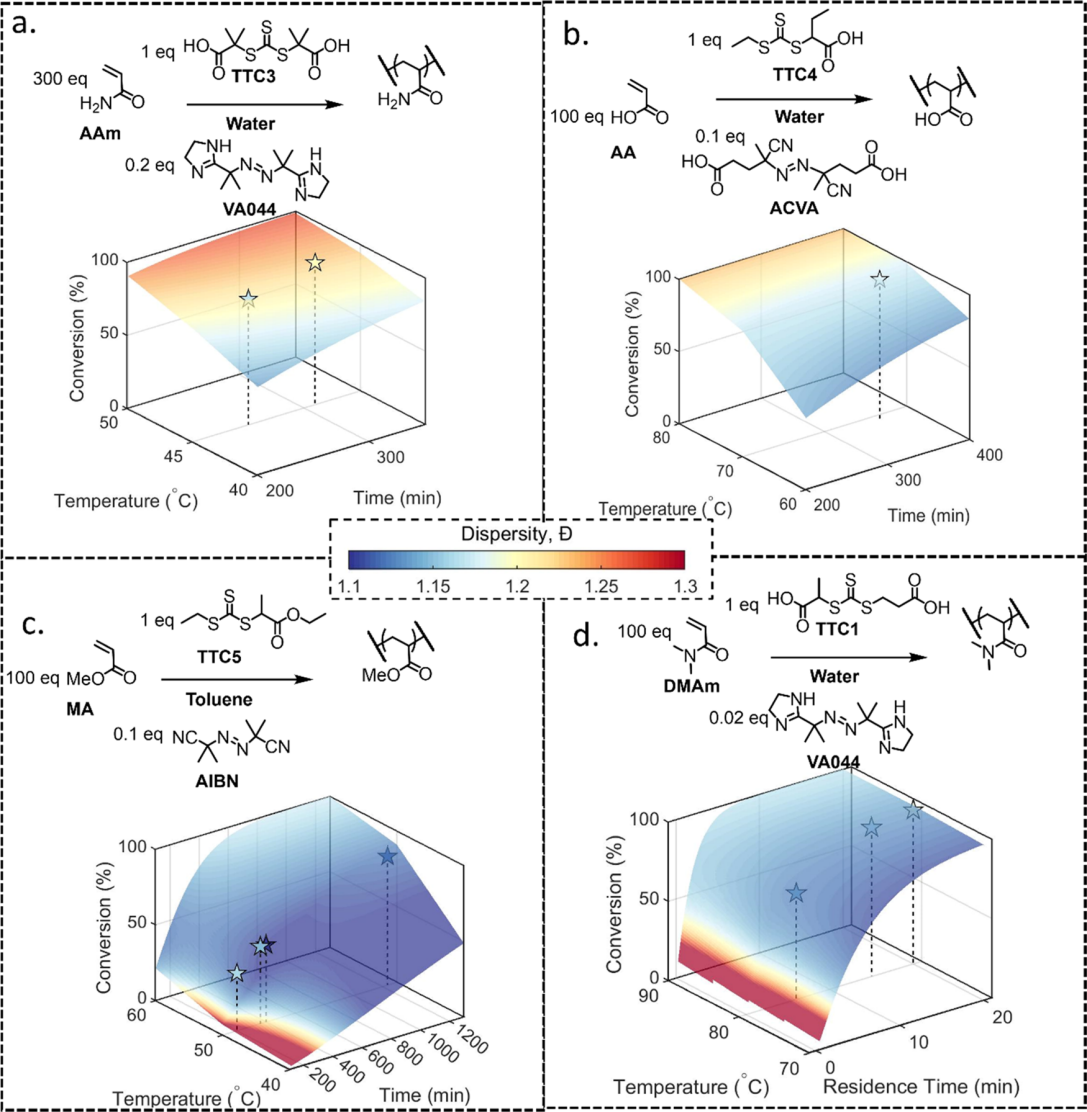
*In silico* kinetic surfaces with literature data (stars) overlayed for the polymerization of (a) AAm,^73^ (b) AA,^75^ (c) MA,^74^ and (d) DMAm (this work). The color of the star and surface corresponds to dispersity (see color bar). Experimental literature data for AAm were reproduced from Liang *et al*.^73^ with permission from Springer, copyright 2017. Experimental data for AA was reproduced from Ji *et al*.^75^ with permission from Taylor and Francis, copyright 2010. Experimental data for MA was reproduced from Wood *et al*.^74^ with permission from CSIRO, copyright 2007.

Broad agreement between the literature data and simulation was observed (Figure 7). Deviations for both conversion and dispersity were limited (see Table 3) for AAm and AA. For AAm, the influence of initiator can be observed; as the initiator concentration is increased, the reaction takes less time to reach high conversion. This is also reflected in the simulated dispersity, the increased radical concentration increases the number of terminative events leading to broader MWDs, which is reflected in the fourth term of the equation. A systematic underestimation of conversion was observed for MA, which could be attributed to a lower concentration of solids (10 w/w %)^76^ used for the rate constant measurement compared to the experimental data (30 w/w %),^74^ or the neglection of side reactions that increase the concentration of propagating species, as has been shown for methylated acrylamide monomers.^62^

For monomers such as acrylamides and acrylic acid and less bulky acrylates, the equation and model work well; however, due to the absence of backbiting and cross-termination effects, the model will fail for bulky acrylates. At high temperatures > 120 °C, the model will become invalid due to the formation of macromonomers and β-scission, which is shown in the literature to cause rate retardation and broadening of the MWD. In addition, for less compatible RAFT systems such as use of MAMs with dithiobenzoate RAFT agents where the retardation is more significant, the degeneracy assumption will not be sufficient and the model will again become invalid. Thus, the model only will work for controlled systems.

## Conclusions

A combined model has been designed to enable computational simulation of the RAFT polymerization process for the purpose of guiding an automated platform. This combines an effective model for conversion, which could be implemented under isothermal conditions, or under polythermal conditions, where the simulation can take into account varying temperature. These were both validated by conducting the RAFT polymerization of DMAm in a flow reactor (operating near-isothermally due to efficient heat transfer) or a batch reactor, where a previously recorded temperature profile was used in the simulation.

The model for predicting dispersity as a function of the conversion was derived based on block-and-blend theory, with the addition of a novel fourth term quantifying the contribution of the terminative events at higher conversion. This results in an upturn in the dispersity at high conversion, which is typically seen in RAFT polymerization.

Finally, for simulating the outcome of reactions in a flow reactor, it was necessary to add a term to account for the contribution of the RTD to the molar mass dispersity. The conversion and dispersity models and the option for an RTD correction (for flow reactors) were programmed into a computational package that enabled prediction of the outcome of RAFT polymerization using trithiocarbonate RAFT agents for monomers with known *k*_p_. Validation of the model was performed in flow, where the experimental values for conversion and dispersity were in good agreement. Furthermore, the model is also in good agreement with several examples from the literature. Although it is recognized that models may not always reflect the exact polymerization process, it provides an opportunity to better predict the outcome of a RAFT polymerization reaction which can be used to guide an automated reactor, potentially streamlining closed-loop self-optimization systems, which previously had no prior knowledge of the chemistry.

## Data Availability

All data supporting this study are provided as Supporting Information accompanying this paper. A full derivation and predictive Excel spreadsheet is available in the Supporting Information, and an application containing both models is also available in the SI. The full equation can be implemented in a MATLAB application, which is available on GitHub: https://github.com/ClarissaYPWilding/KineticsModellerRAFT or an Excel spreadsheet, which allows calculation of the dispersity using both analytical and Gaussian quadrature method available free of charge in the Supporting Information.

## Supporting Information Available

Analytical derivation of rate equations; dispersity derivation; simulated data comparing parameters; residence time distribution; and rate constants (PDF)
RAFT dispersity calculator (ZIP)
