# Supplementary material for: Development and Experimental Validation of a Dispersity Model for In Silico RAFT Polymerization
Source: Macromolecules. 2023 Feb 9;56(4):1581–91. doi: 10.1021/acs.macromol.2c01798 (PMC9979647; doi:10.1021/acs.macromol.2c01798)
Supplement: Supplementary file 1 — ma2c01798_si_001.pdf [file ma2c01798_si_001.pdf]

Supplementary Information

Development and Experimental Validation of a Dispersity Model for In-silico RAFT Polymerization

Clarissa. Y. P. Wilding[a,b], *Stephen. T. Knox*[a,b], *Richard. A. Bourne*[a,b] *and Nicholas. J. Warren*[a,b] *

a. School of Chemical and Process Engineering, University of Leeds, LS2 9JT

b. Institute of Process Research and Development, School of Chemistry, University of Leeds, LS2 9JT

** Author to whom correspondence should be addressed*

Contents

1. **Analytical Derivation of Rate Equations**[1–3] 2
2. **Dispersity Derivation** 3
3. **Simulated data comparing parameters** 11
4. **Residence time distribution** 12
5. **Experimental** 16
6. **Rate Constants** 18

1. Analytical Derivation of Rate Equations[1-3]

The following equations are also found in the main script

$\frac{d[P_r]}{dt} = r_{Ini} + k_\beta[CTA_a] - k_a[P_r][CTA] - 2k_t[P_r]^2 - k_{ct}[P_r][CTA_a]$ *[S 1]*

$\frac{d[CTA_{adduct}]}{dt} = k_a[P_r][CTA] - k_\beta[CTA_a] - k_{ct}[P_r][CTA_a]$ *[S 2]*

$\frac{d[CTA]}{dt} = k_\beta[CTA_a] - k_a[P_r][CTA]$ *[S 3]*

$\frac{d[P]}{dt} = k_t[P_r]^2 + \frac{1}{2}k_t[P_r]^2$ *[S 4]*

$\frac{d[P']}{dt} = k_{ct}[P_r][CTA_a]$ *[S 5]*

Assuming steady state kinetics, $\frac{d[CTA_{adduct}]}{dt} = 0$, so the concentration of the Dormant species is constant. Consequently, [S 2] can be simplified down to [S 6]

$0 = k_a[P_r][CTA] - k_\beta[CTA_a] - k_{ct}[P_r][CTA_a]$ *[S 6]*

If the radical adduct concentration does not change there has to be a constant rate of cross termination so $0 = k_a[P_r][CTA] - k_\beta[CTA_a]$, allowing a simplified equation in the form of

$[CTA_{adduct}] = \frac{k_a}{k_\beta}[P_r][CTA]$ [S 7]

Rate of radical transfer from the CTA adduct species to form the 3 armed polymer species is assumed to be constant at steady state so that, $\frac{d[CTA_a]+d[P_r]}{dt} \approx 0$, [S 2] and [S 1] -2can be combined and simplified down. This allows an equation for [R] at steady state to be formulated from [S 8] to [S 9]

$\frac{d[CTA_a]+d[P_r]}{dt} = k_a[P_r][CTA] - k_\beta[CTA_a] - k_{ct}[P_r][CTA_a] + r_{Ini} + k_\beta[CTA_a] - k_a[P_r][CTA] - 2k_t[P_r]^2 - k_{ct}[P_r][CTA_a]$ *[S 8]*

$$0 = r_{Ini} - k_t[P_r]^2 - 2k_{ct}[P_r][CTA_a]$$

$$r_{Ini} = k_t[P_r]^2 + 2k_{ct}[P_r][CTA_a]$$

r_I is the initiation rate.

$[P_r] = \sqrt{\frac{r_{Ini}}{k_t + 2[CTA]\frac{k_a k_{ct}}{k_\beta}}}$ *[S 9]*

Combining [S 3] with [S 2] when $\frac{d[CTA_a]}{dt} = 0$ can be used to find the rate of change of [CTA], while accounting for the equilibrium [S 10] obtained.

$$\frac{d[CTA]}{dt} + \frac{d[CTA_a]}{dt} = k_\beta[CTA_a] - k_a[P_r][CTA] + (k_a[P_r][CTA] - k_\beta[CTA_a] - k_{ct}[P_r][CTA_a])$$

$$\frac{d[CTA]}{dt} = -k_{ct}[P_r][CTA_a] \quad [S\ 10]$$

The algebraic values for $[P_r]$ $and\ CTA_a$ can be substituted in to [S 10] to form

$$= k_{ct}\left(\sqrt{\frac{r_{Ini}}{k_t+2[CTA]\frac{k_a k_{ct}}{k_\beta}}}\right)^2 \left(\frac{k_a}{k_\beta}\right)[CTA]$$

Simplify further $\frac{d[CTA]}{dt} = -\,k_{ct}\left(\frac{r_{Ini}}{k_t+2[CTA]\frac{k_a k_{ct}}{k_\beta}}\right)\left(\frac{k_a}{k_\beta}\right)[CTA]$

$$\frac{d[CTA]}{dt} = \left(\frac{-\,k_{tr}\,r_{Ini}\,k_a[CTA]}{(k_t+2[CTA]\frac{k_a k_{ct}}{k_\beta})k_\beta}\right) \quad [S\ 11]$$

$$\frac{d[CTA]}{dt} = \left(\frac{-\,k_{ct}\,r_{Ini}\,k_{add}[CTA]}{k_t k_\beta + 2[CTA]k_a k_{ct}}\right)$$

Finally, an equation for the change in concentration of CTA vs time can be reduced and solved using MATLAB by taking the initial $[CTA]_0$ as the t=0 value.

$$\frac{d[CTA]}{dt} = -\frac{r_{Ini}}{\frac{k_t k_\beta}{k_{ct}k_a[CTA]}+2} \quad [S\ 12]$$

Following this we can then solve [S 9] as we have a value for [CTA] vs time. This gives us the $[P_r]$ at steady state at a given time.

2. Dispersity Derivation

Assumptions of the model

- Under a quasi-equilibrium state
- The equilibria can be described by the partition coefficient

$$k_{tr} = k_a\phi = k_a\left(\frac{k_\beta}{k_{-a}+k_\beta}\right) \quad [S\ 13]$$

$$k_{-tr} = k_{-\beta}(1-\phi) = \frac{k_{-a}}{k_{-a}+k_\beta} \quad [S\ 14]$$

- Rate constants are only dependent on temperature following Arrhenius
- Chain-length dependent termination is only accounted for in the termination parameter.
- Self-initiation, quenching and the formation/decomposition of intermediate radical adduct is assumed to be fast.
- Solvent effects are only accounted for in the solution propagation rate constant

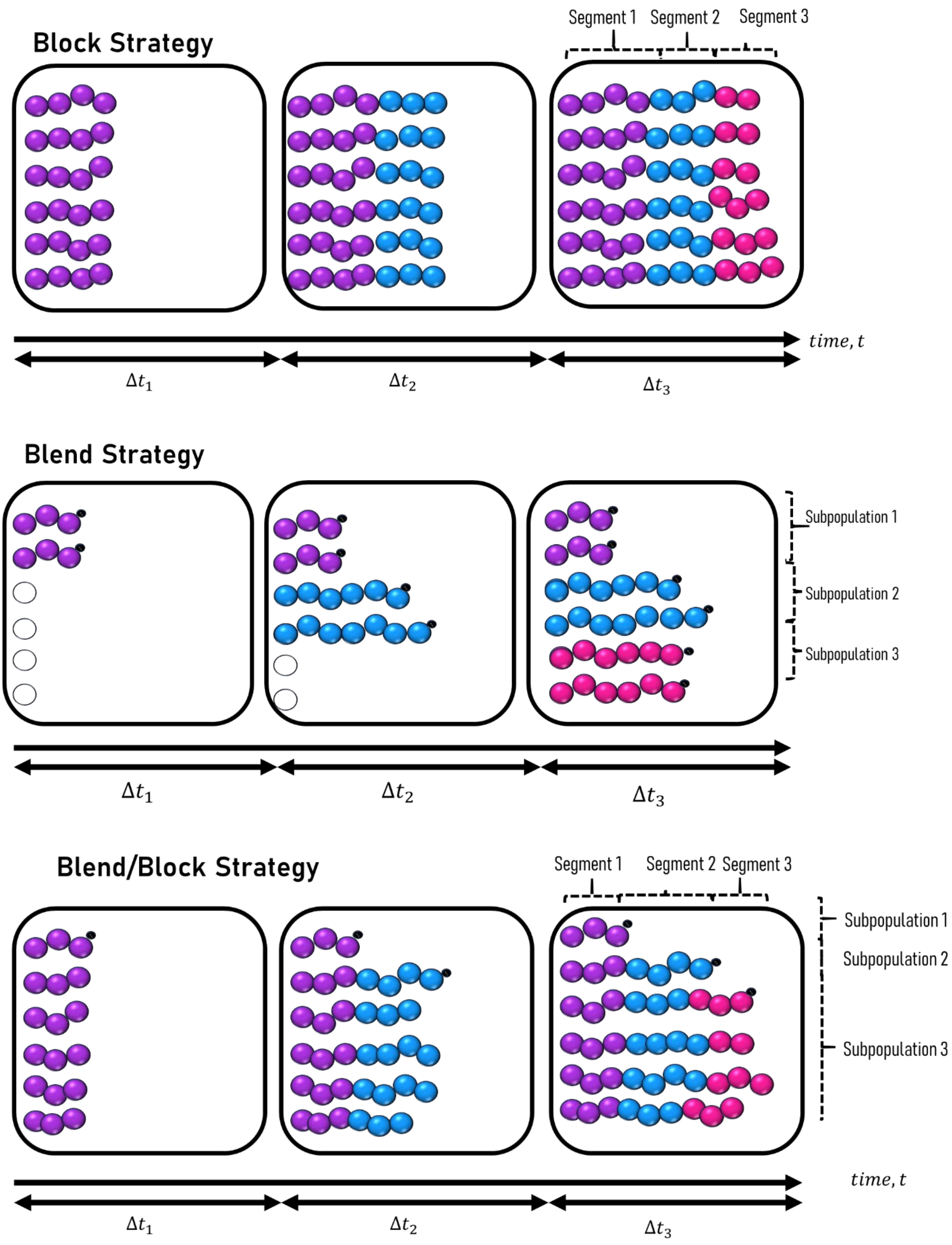

Figure S1. Schematic depiction of combining blend and block theory

$$r_n = L_n = \frac{[M]_0 \alpha}{[CTA]_0} \qquad [S\ 15]$$

Firstly, a term to describe the probability of propagation in each RAFT equilibrium cycle is defined as, ρ_p. the ratio of propagation of active polymer chains to all steps using propagating radicals.

$$\rho_p = \frac{k_p[M]}{k_p[M]+k_{tr}[CTA]+k_t[P_r]} \qquad [S\ 16]$$

Where the overall rate constants for the equilibrium is described by [S 13] and [S 14] .[4] Approximate

k_a, and ϕ can be found in the literature.

The expected number of transfer cycles $\Delta\tau_i$ for each segment, i, can be defined as

$$\Delta\tau_i = k_{-tr}\frac{[P_r][CTA\}}{[CTA]} = k_{-tr}[P_r]\Delta t_i \qquad [S\ 17]$$

The expected number of monomer units added in each cycle can be given by

$$x = \frac{\rho_p}{1-\rho_p} = \frac{k_p[M]}{k_{tr}[CTA]+k_t[P_r]} = \frac{k_p[M]}{k_{tr}[CTA]}\ when\ (k_{tr}[CTA] \gg k_t[P_r]) \qquad [S\ 18]$$

$$r_{n,i} = \frac{k_p[M]_i k_{-tr}}{k_{tr}[CTA]_i}\Delta t_i \qquad [S\ 19]$$

$$Đ_i = 1 + \frac{1}{r_{n,i}} + \frac{2}{k_{-tr}\,\Delta t_i} \qquad [S\ 20]$$

Where $r_{n,i}$ is the degree of polymerization of each block represented by the product of the expected number of transfer cycles and the expected number of monomeric units added per cycle. $[M]_0$ is the monomer concentration at t =0, α is the conversion, M_{Mr} is the molecular mass of the monomer and $[CTA]_0$ is the concentration of CTA at time = 0. The equation for $Đ_i$ was previously derived by Mastan *et al.*[5]

$$r_n^{block} = \sum_{i=1}^{N} r_{n,i} = \lim_{\Delta t_i\to 0}\sum_{i=1}^{N}\frac{k_p[M]_i k_{tr}[P_r]}{k_{-tr}[CTA]_i}\Delta t_i = \int_0^{\alpha}\frac{k_p[M]k_{tr}}{k_{-tr}[CTA]}da = \frac{[M]_0\alpha}{[CTA]_0} \qquad [S\ 21]$$

To rewrite as a function of conversion the equation for monomer conversion can be rewritten and rearranged to S 22

$$\frac{d\alpha}{dt} = k_p(1-\alpha)[P_r] \rightarrow rearrange \rightarrow dt = \frac{d\alpha}{k_p(1-\alpha)[P_r]} \qquad [S\ 22]$$

$$Đ^{block} = 1 + \frac{1}{r_n^{block^2}}\sum_{i=1}^{N}\left(r_{n,i}\right)^2(Đ_i - 1) \qquad [S\ 23]$$

Propagating chains terminated in each Δt_i are discretized into their sub populations. As in controlled polymerizations, chains that are growing can terminate, forming blocks, therefore blend strategy is applied.

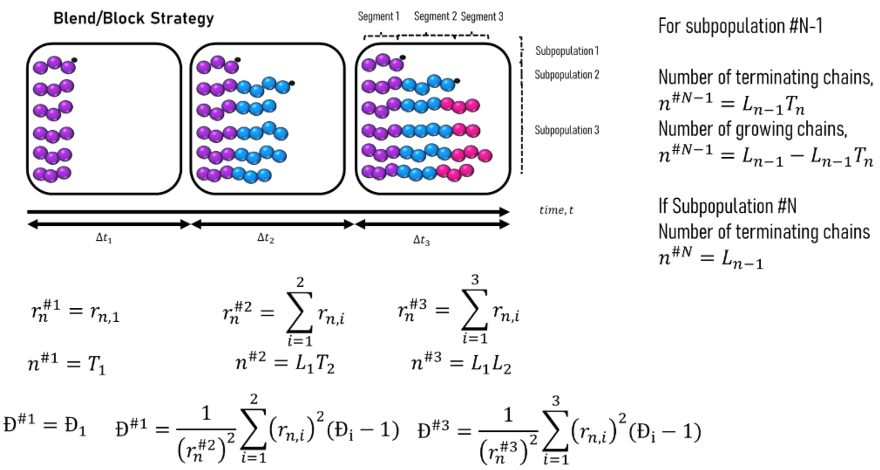

Figure S2. Contributions of termination and livingness in blend and block theory.

For example, in subpopulation 1 there is 1 terminating chain so, $n^{\#1} = T_1$, in subpopulation 2 segments 1 and 2 grow so $n^{\#2} = L_1T_2$ and in subpopulation 3, the number of growing chains is 2 so $n^{\#3} = L_1L_2$.

T is the number fraction of dead chains whereas L is the number fraction of Living chains.

The number of living chains is described by the ratio of $\frac{[CTA]_t}{[CTA]_0}$ [6] the number fraction of dead chains can be described by the ratio of polymer to initial RAFT agent

As in Mastan et al.[7] the following equations can be produced for RAFT.

$$r_n^{blend} = \sum_{i=1}^{N} n_i r_{n,i}^{block} = \frac{[M]_0}{[CTA]_0}\alpha \qquad [S\ 24]$$

$$Đ^{blend} = \frac{1}{(r_n^{blend})^2}\sum_{i=1}^{N} n_i (r_{n,i}^{block})^2\, Đ_i^{block} \qquad [S\ 25]$$

By substituting $Đ_i$ from [S 23] and $r_{n,i}^{block}$ from [S 21] into [S 25]

$$Đ = 1 + \frac{1}{(r_n)^2}\lim_{\Delta t_i \to 0}\sum_{i=1}^{N} L_{i-1}^{O}\,(r_{n,i})^2(Đ_i - 1) + \frac{2}{(r_n)^2}\lim_{\Delta t_i \to 0}\sum_{i=1}^{N} L_{i-1}^{O}\, r_{n,i}\sum_{j=1}^{i} T_{j-1}^{O}\, r_n, j \qquad [S\ 26]$$

$$\sum_{i=1}^{N} r_{n,i} = \lim_{\Delta t_i \to 0}\sum_{i=1}^{N}\frac{k_p[M]_i[P_r]}{k_{tr}[CTA]_i}\Delta t_i = \int_0^t \frac{k_p[M]k_{-tr}[P_r]}{k_{tr}[CTA]}dt = \int_0^\alpha \frac{k_p[M]_i\, k_{-tr}[P_r]}{k_{tr}[CTA]}\frac{da}{k_p(1-\alpha)[P_r][P_r]} = \int_0^\alpha \frac{[M]_i[M]_0}{[CTA]_i}\frac{d\alpha}{([M]_i)} = \frac{[M]_0}{[CTA]_0}\alpha \qquad [S\ 27]$$

A. **Terms 1-3**

1. **Substitute in $r_{n,i}$, $L^{O}{}_{i-1}$ *and* $Đ_i$**

$$T_{1-3} \equiv 1 + \frac{[CTA]_0^2}{[M]_0^2\alpha^2}\frac{[CTA]}{[CTA]_0}\lim_{\Delta t_i \to 0}\sum_{i=1}^{N}\left(\frac{k_p[M]k_{-tr}[P_r]}{k_{tr}[CTA]}\Delta t_i\right)^2\left(\frac{1}{\frac{k_p[M]k_{-tr}[P_r]}{k_{tr}[CTA]}\Delta t_i} + \frac{2}{k_{-tr}[P_r]\Delta t_i}\right) \quad [S\ 28$$

2. **T_{1-3} shown in [S 28] can be simplified down to [S 30]**

$$T_{1-3} \equiv 1 + \frac{[CTA]_0^2}{[M]_0^2\alpha^2}\frac{[CTA]}{[CTA]_0}\lim_{\Delta t_i \to 0}\sum_{i=1}^{N}\left(\frac{k_p[M]k_{-tr}[P_r]}{k_{tr}[CTA]}\Delta t_i + \frac{2k_p^2[M]_i^2k_{-tr}^2[P_r]^2\,\Delta t_i^2}{k_{tr}^2[CTA]_i{}^2k_{-tr}\Delta t_i}\right) \qquad [S\ 29]$$

$$T_{1-3} \equiv 1 + \frac{[CTA]_0^2}{[M]_0^2\alpha^2}\frac{[CTA]}{[CTA]_0}\lim_{\Delta t_i \to 0}\sum_{i=1}^{N}\left(\frac{k_p[M]k_{-tr}[P_r]}{k_{tr}[CTA]}\Delta t_i\right)^2\left(\frac{k_p[M]k_{-tr}[P_r]}{k_{tr}[CTA]}\Delta t_i + \frac{2k_p^2[M]_i^2k_{-tr}^2[P_r]\Delta t_i^2}{k_{tr}^2[CTA]_i{}^2k_{-tr}\Delta t_i}\right) \qquad [S\ 30]$$

3. The expression can then be split up into 3 terms shown in [S 32] and [S 33]

$$Assumption\colon \lim_{\Delta t_i \to 0}\sum_{i=1}^{N}\frac{k_p[M]_ik_{-tr}[P_r]}{k_{tr}[CTA]_i}\Delta t_i = \int_0^t\frac{k_p[M]k_{-tr}[P_r]}{k_{tr}[CTA]}dt$$

$$T_{1-3} \equiv 1 + \frac{[CTA]_0^2}{[M]_0^2\alpha^2}\lim_{\Delta t_i \to 0}\sum_{i=1}^{N}\frac{k_p[M]k_{-tr}[P_r]}{k_{tr}[CTA]}\Delta t_i + \frac{[CTA]_0^2}{[M]_0^2\alpha^2}\lim_{\Delta t_i \to 0}\sum_{i=1}^{N}\frac{2k_p[M]_i}{k_{tr}[CTA]_i}\frac{k_p[M]_ik_{-tr}[P_r]}{k_{tr}[CTA]_i}\Delta t_i \qquad [S\ 31]$$

$$T_{1-3} \equiv 1 + \frac{[CTA]_0^2}{[M]_0^2\alpha^2}\int_0^t\frac{k_p[M]k_{-tr}[P_r]}{k_{tr}[CTA]}dt + \frac{[CTA]}{[CTA]_0}\frac{[CTA]_0^2}{[M]_0^2\alpha^2}\int_0^t\frac{2k_p[M]_0}{k_{tr}[CTA]_i}\frac{k_p[M]_ik_{-tr}[P_r]}{k_{tr}[CTA]_i}dt \qquad [S\ 32]$$

4. Put Expression in terms of conversion using assumption described by [S 22] then simplify down further to [S 33]

$$T_{1-3} \equiv 1 + \frac{[CTA]_0^2}{[M]_0^2\alpha^2}\int_0^\alpha\frac{[M]_0}{[CTA]_i}d\alpha + \frac{[CTA]}{[CTA]_0}\frac{[CTA]_0^2}{[M]_0^2\alpha^2}\int_0^\alpha\frac{2k_p[M]_0}{k_{tr}[CTA]_i}\frac{[M]_0}{[CTA]_i}d\alpha$$

$$T_{1-3} \equiv 1 + \frac{r_n}{r_n^2} + \frac{[CTA]}{[CTA]_0}\frac{[CTA]_0^2}{[M]_0^2\alpha^2}\int_0^x\frac{2k_p(1-x)}{k_{tr}[CTA]_i}\frac{[M]_0^2}{[CTA]_i}d\alpha$$

$$T_{1-3} \equiv 1 + \frac{1}{r_n} + \frac{2k_p}{k_{tr}}\frac{[M]_0^2}{[CTA]}\frac{[CTA][CTA]_0}{[M]_0^2\alpha^2}\int_0^\alpha\frac{(1-\alpha)}{[CTA]_i}d\alpha$$

$$T_{1-3} \equiv 1 + \frac{1}{r_n} + \frac{k_p[CTA]_0}{k_{tr}}\frac{2}{\alpha^2}\int_0^a\frac{(1-\alpha)}{[CTA]_i}da \qquad [S\ 33]$$

$$Đ = 1 + \frac{1}{r_n} + \frac{k_p[CTA]_0}{k_{tr}}\frac{2}{\alpha^2}\int_0^a\frac{(1-\alpha)}{[CTA]_i}da + T_4 \qquad [S\ 34]$$

B. Term 4 – Quantifying Terminative events

Substitute in $r_{n,i}$, $L^O{}_{i-1}$, $Đ_i$, $r_{n,j}$, and T_{j-1}^O

Where the termination fraction, T^o is approximated as the ratio of polymer to CTA multiplied by DP[S 35]

$$T_{j-1}^O \approx \frac{[P]}{[CTA]} \qquad [S\ 35]$$

$$T_4 \equiv 2\frac{[CTA]_0^2}{[M]_0\alpha^2}\lim_{\Delta t_i \to 0}\sum_{i=1}^{N}\frac{[CTA]_i}{[CTA]_0}\left(\frac{k_{-tr}[P_r]k_p[M]_i}{k_{tr}[CTA]_0}\right)\Delta t_i\ \sum_{j=1}^{i}\frac{[P]}{[CTA]}\left(\frac{k_{-tr}[P_r]k_p[M]_i}{k_{tr}[CTA]_0}\Delta t_i\right) \qquad [S\ 36]$$

$$T_4 \equiv 2\frac{[CTA]_0^2}{[M]_0^2\alpha^2}\int_0^t\frac{[CTA]_i}{[CTA]_0}\left(\frac{k_{-tr}[P_r]k_p[M]_i}{k_{tr}[CTA]_0}\right)dt\int_0^t\frac{[P]}{[CTA]}\left(\frac{k_{-tr}[P_r]k_p[M]_i}{k_{tr}[CTA]_0}dt\right)$$

$$T_4 \equiv 2\frac{[CTA]_i[CTA]_0}{[\mathrm{M}]_0\alpha^2}\int_0^t\frac{k_{-tr}[P_r]k_p[M]_i}{k_{tr}[CTA]_0}dt\int_0^t\frac{[P]}{[CTA]}\frac{k_{-tr}[P_r]k_p[M]_i}{k_{tr}[CTA]_0}dt$$

$$T_4 \equiv 2\frac{[CTA]_i[CTA]_0}{[\mathrm{M}]_0\alpha^2}\int_0^a\frac{k_{-tr}[P_r]k_p[M]_i}{k_{tr}[CTA]_0}\frac{da}{k_p\left(\frac{[M]_i}{[M]_0}\right)[P_r]}\quad\int_0^a\frac{[P]}{[CTA]}\cdot\frac{k_{-tr}[P_r]k_p[M]_i}{k_{tr}[CTA]_0}\frac{da}{k_p\left(\frac{[M]_i}{[M]_0}\right)[P_r]}$$

$$T_4 \equiv 2\frac{[CTA]_i[CTA]_0}{[\mathrm{M}]_0\alpha^2}\int_0^a \frac{k_{-tr}[P_r]k_p[M]_i}{k_{tr}[CTA]_0}\frac{da[M]_0}{k_p[M]_i[P_r]}\int_0^a \frac{[P]}{[CTA]}\cdot\frac{k_{-tr}[P_r]k_p[M]_i}{k_{tr}[CTA]_0}\frac{da\,[M]_0}{k_p[M]_i[P_r]}$$

$$T_4 \equiv 2\frac{[CTA]_i[CTA]_0}{[\mathrm{M}]_0\alpha^2}\int_0^a \frac{[M]_0}{[CTA]_i}da \ \int_0^a \frac{[P]}{[CTA]}\cdot\frac{[M]_0}{[CTA]_i}da$$

$$T_4 \equiv \frac{2}{\alpha^2}\int_0^\alpha (\int_0^a \frac{[P]}{[CTA]}da) \quad d\alpha \qquad [S\ 37]$$

By substituting [S 35] and [S 22], an equation for dispersity in terms of the integrals [S 37] can be obtained as a function of the conversion.

$$Đ = 1 + \frac{1}{DP} + \frac{2}{X^2}\frac{k_p}{k_{tr}}[CTA]_0\int_0^\alpha \frac{1-a}{[CTA]}da + \frac{2}{X^2}\int_0^\alpha (\int_0^a \frac{[P]}{[CTA]}da)\ da \qquad [S\ 38]$$

An Ideal RAFT System

[S 33] which approximates the number of monomers added per cycle can be separated, analytically integrated, and simplified. If we assume that the overall concentration of [CTA] does not change over time. $[CTA]_0 = [CTA]$ Then [S 39] can become [S 40]

$$T_3 \equiv \frac{2}{\alpha^2}\frac{k_p}{k_{tr}}\frac{[CTA]_0}{[CTA]}\left[a - \frac{1}{2}a^2\right]_0^\alpha \qquad [S\ 39]$$

$$T_3 \equiv \frac{k_p}{k_{tr}}\left(\frac{2}{X} - 1\right) \qquad [S\ 40]$$

Formulating an approximate equation for the number of dead chains

[S 35], which is the approximation of the dead chain contribution can be simplified before integrating with Taylor expansion followed by Gaussian quadrature.

$[P]$, can be found by integrating the rate equation for the formation of polymer [S 41] with respect to time to obtain [S 43]

$$\frac{d[P]}{dt} = k_t[P_r]^2 \qquad [S\ 41]$$

$$[P] = k_t[P_r]^2 t \qquad [S\ 42]$$

Where α, or conversion can be described by the decay in monomer concentration, assuming all monomer is converted to polymer as $\alpha = 1 - x$, where x is the ratio of monomer left in the polymerization to the initial monomer concentration ($x = \frac{[M]_t}{[M]_0}$)

RAFT exhibits pseudo first order kinetics with respect to monomer conversion as shown by [S 43], this can be rewritten in terms of the ratio of monomer left in the reaction shown by [S 44]

$$\frac{d[M]}{dt} = -k_p[M][P_r] \qquad [S\ 43]$$

$\frac{dx}{dt} = k_p(1-x)\sum[P_r]$ *[S 44]*

[S 44] can be rearranged and integrated analytically to find the relationship between conversion and time.

$t = -\frac{ln(1-\alpha)}{k_p[P_r]}$ *[S 45]*

The expression for T_4 in can then be simplified in terms of conversion, α, using [S 45] and [S 44] to give [S 46]

$T_4 = \frac{2}{X^2[CTA]_0}(\int_0^{\alpha}(\int_0^{\alpha}\frac{k_t[P_r]\,ln(1-\alpha)}{k_p[CTA]}da)\,d\alpha)$ *[S 46]*

The rate of initiation can be given as, r_{Ini}, (shown in S 47)which produces the initiating radicals, at time = 0 this is $r_{Ini} = 2k_d f[I]_0$. To account for the regeneration of radicals the rate of formation of single monomer radicals is used as the radical forming step, $-\frac{d[M]}{dt} = \frac{d[M\cdot]}{dt} = k_p[M][M\cdot]$. If the number of monomeric radicals does not change and is dependent on the rate of initiation.

$r_{Ini} = 2k_d f[I]_0 e^{-k_d t}$ *[S 47]*

$r_R = k_p[M][M\cdot]$ *[S 48]*

Where $[M\cdot] = \sqrt{\frac{r_{Ini}}{2k_t}}$ if we assume that As RAFT includes initiation and termination steps these are accounted for by including r_{Ini} and k_t as additional terms in the concentration of propagating radicals. If we assume the RAFT equilibrium is degenerate, then $k_{tr} = k_{-tr}$

$\frac{d[P_r]}{dt} = r_R - k_{tr}[CTA]_x[P_r] + k_{-tr}[CTA]_y[P_r] - 2k_t[P_r]^2 = 0$ *[S 49]*

Mathematical solvation of this quadratic for $[P_r]$ returns 2 solutions a positive and a negative. However, we know we can only use the positive solution as Propagating radicals are generated. This is further simplified to [S 50]

$\frac{d[P_r]}{dt} = r_R - 2k_t[P_r]^2$ *[S 50]*

We then simplify the initial rate (at t=0) of radical production by assuming that the rate is also dependent on propagation and termination we can get an approximate value for r_R. By assuming that radicals are produced instantaneously at t=0 and the initial rate of propagating radical production will be approximately the initial rate of polymerization as $-\frac{d[M]}{dt} = \frac{d[M\cdot]}{dt}$

$\gamma \approx \frac{2}{\alpha^2}\frac{k_t}{k_p[CTA]_0}\sqrt{\frac{r_R}{2k_t}}$ *[S 51]*

Tackling the double integral, I. The double integral in S 52 is solved analytically in MATLAB using the symbolic math toolbox to give S 53 which can then be simplified and tidied down to S 54. .

$$I = \int_0^{\alpha}\int_0^{\alpha} -ln(1-a)\, da \qquad [S\ 52]$$

$$I = (ln(1-\alpha)(\alpha^2-1) - (\alpha-1)(ln(1-\alpha)-1) + 2\alpha + \frac{3\alpha^2}{4} - 1 \qquad [S\ 53]$$

$$I = \frac{2(ln(1-\alpha)(\alpha^2-2\alpha+1)+(2-3\alpha)\alpha}{4}$$

$$= \frac{2(ln(1-\alpha)(\alpha^2-1)+(2-3\alpha)\alpha}{4} \qquad [S\ 54]$$

The logarithm can then re-written as a Taylor series as in S 55

$$T_4 = \frac{2}{\alpha^2}\frac{k_t}{k_p}\sqrt{\frac{\mathrm{r_R}}{2k_t}}.I$$

$$T_4 = \frac{k_t}{k_p[CTA\}_0}\sqrt{\frac{r_R}{2k_t}}\left\{\frac{(2-3\alpha)+}{2\alpha} + \frac{(\alpha-1)^2}{\alpha^2}\right\}\left(-\alpha - \frac{\alpha^2}{2} - \frac{\alpha^3}{3} - \frac{\alpha^4}{4} - \cdots\right) \qquad [S\ 55]$$

Finally, S 55 is expanded and only the first 2 taylor terms are used to give S 56

$$T_4 \approx \frac{k_t}{k_p[CTA]_0}\sqrt{\frac{r_R}{2k_t}}\left(\frac{\alpha}{3} + \frac{\alpha^2}{12}\right) \qquad [S\ 56]$$

$$Đ = 1 + \frac{1}{DP} + \frac{k_{tr}}{k_p}\left(\frac{2}{\alpha} - 1\right) + \frac{k_t}{k_p[CTA]_0}\sqrt{\frac{r_R}{2k_t}}\left(\frac{\alpha}{3} + \frac{\alpha^2}{12}\right) \qquad [S\ 57]$$

The double integral is solved by Mastan *et al.* by Gaussian quadrature. Then a single term Taylor expansion. The first integral in S 52 is solved to SI 58

$$I = \int_0^{\alpha} \alpha\, ln\left(1 - \frac{a}{2}\right) da \qquad [S\ 58]$$

The second integration converts S 58 to S 59

$$I = \left[a^2\, ln\left(1 - \frac{a}{4}\right)\right]_0^{\alpha} = \alpha^2\, ln\left(1 - \frac{\alpha}{4}\right) \qquad [S\ 59]$$

The full equation for T_4 is given by S 60

$$T_4 = -\frac{k_t}{k_p[CTA]_0}\sqrt{\frac{r_R}{2k_t}}\, ln\left(1 - \frac{\alpha}{4}\right) \qquad [S\ 60]$$

S 60 can then be expressed as a Taylor expansion. In Mastan *et al.* a single Taylor term is used to form S 61

$$T_4 = \frac{k_t}{4k_p[CTA]_0}\sqrt{\frac{r_R}{2k_t}}\,\alpha \qquad [S\ 61]$$

Analytical integration is the more accurate solution, suggesting that there is an assumption in the strategy that leads to higher dispersity than what is experimentally obtained. Despite the analytical method

providing more accurate mathematical approximation the experimental data in both Mastan et al.[7] and Wang[8] the number of assumptions in the simplification of the transfer steps and not accounting for side reactions such as transfer to solvent.

3. Simulated data comparing parameters

Here, we gather rate constants, activation energies, E_a, preexponential factors, A, and efficiency constants from experimental and theoretical literature. E_a and A can be found in the literature for propagation and initiator decomposition which can then be used to calculate k_p and k_d, respectively. Initiator efficiency, f are widely assumed to be between 0.3 and 0.8 for azo initiators, consequently a value of 0.5 has been used. Addition rate constants, k_a was estimated based on https://pubs.acs.org/doi/pdf/10.1021/jp900684t , where it is proposed that the k_a value is around 10^6 orders of magnitude. As widely seen throughout the literature we assume that the basic equilibria seen in the main text lies towards the product such that $K_{eq} = 1000$ for TTCs.

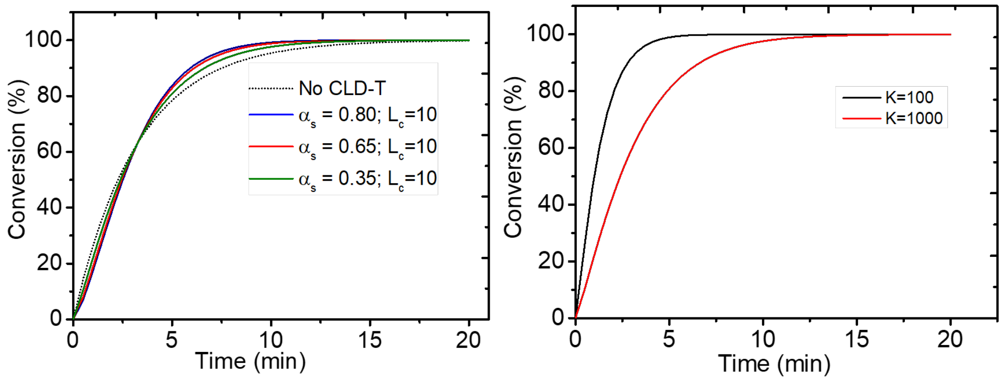

Figure S3. Effect of the CLD-T component on the conversion vs time plot (left), Effect of the ratio of addition to fragmentation on the conversion.

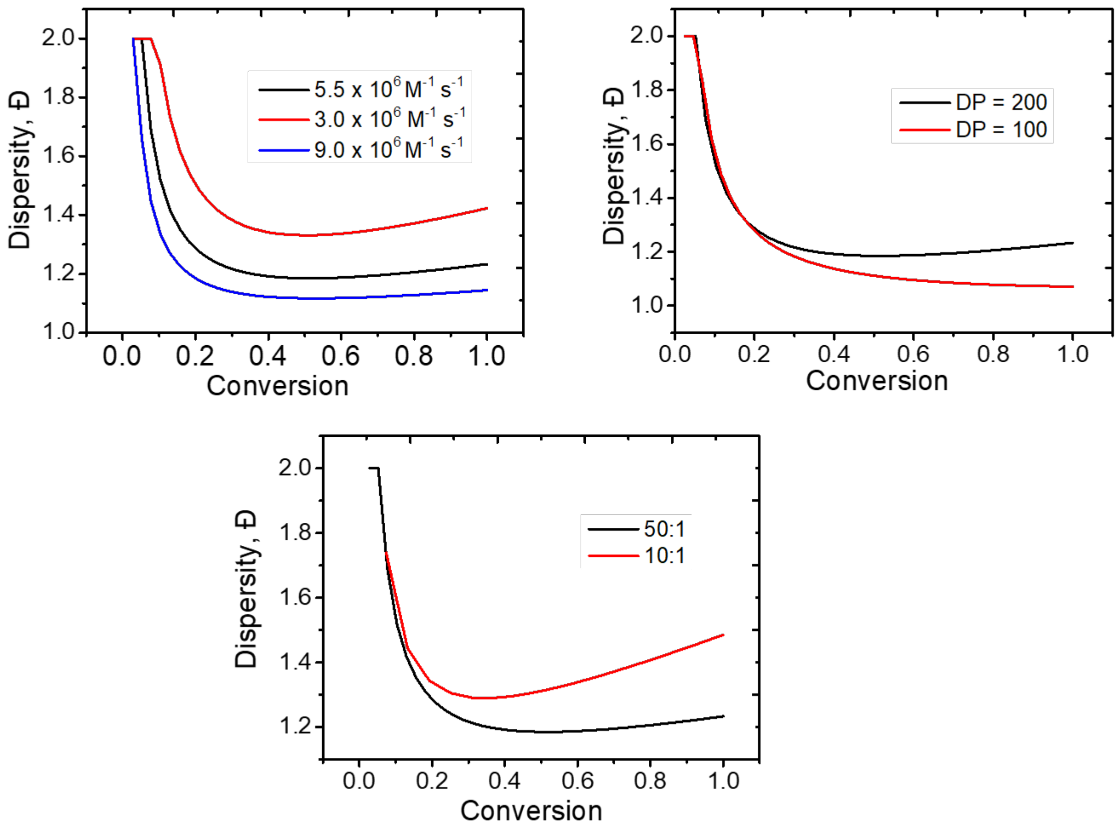

Figure S4. Simulated conversion vs dispersity plots demonstrating the effect of addition rate constant, k_a, the ratio of CTA to initiator and the target degree of polymerization

4. Residence time distribution

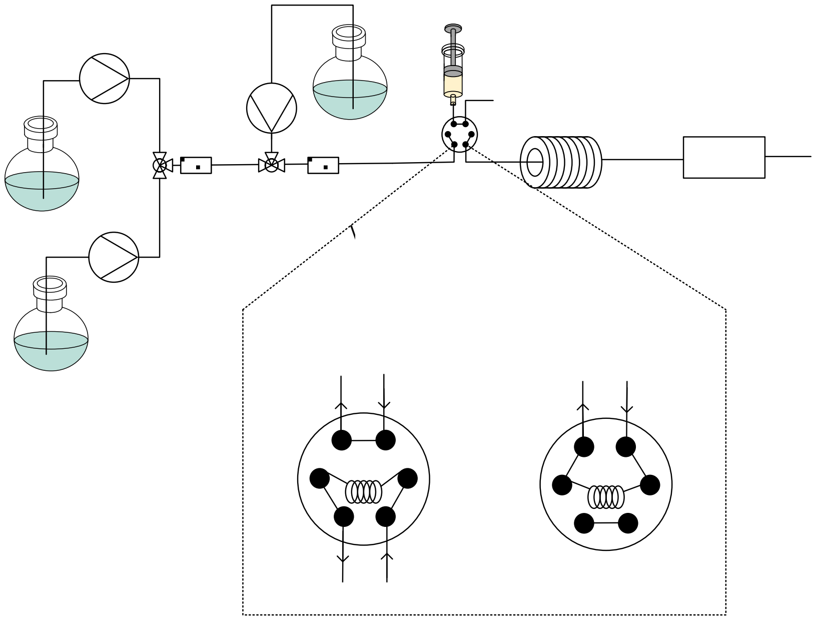

Figure S5. Schematic of the experimental residence time distribution tracer experiment

The residence time distributions of the reactor were characterised using a tracer. The 3 pumps were used to flow solvent through the reactor. A 6-port Vici Valco Cheminert switching valve with EHMA 2 position actuator were used to inject a sample of 30 w/w % reagent solution and polymer solution into the reactor coil as a tracer. The refractive index of the solutions. The RI signals were processed to residence time distribution functions.

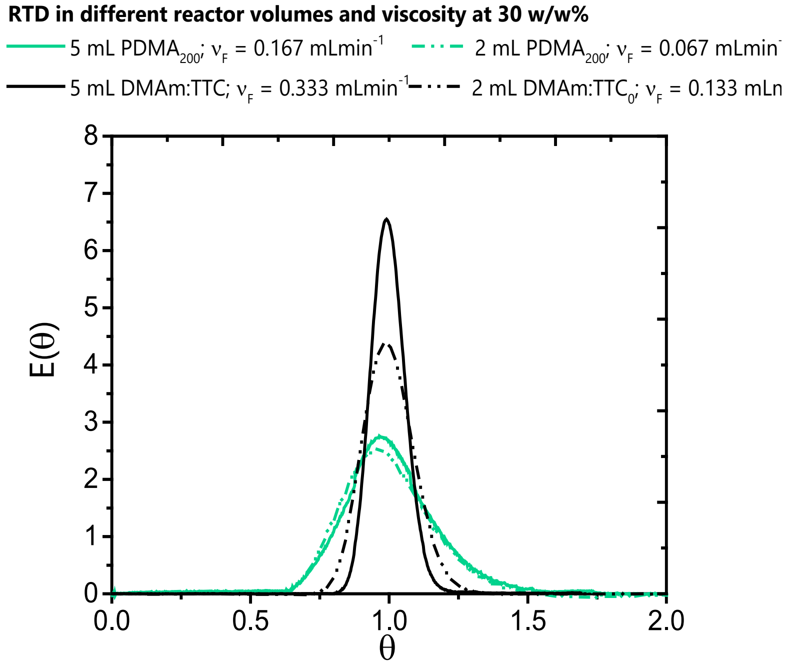

Figure S6. Normalised RTD function plots comparing PDMA200 as a tracer (green) in a 2 mL (dashed-dotted line) and 5 mL (solid line) reactor to DMA200 as a tracer (black) in a 2 mL (dashed-dotted line) and 5 mL (solid line).

It is demonstrated experimentally that the volume of the reactor therefore the flow rates are detrimental to the RTD. The 2mL coil requires slower flowrates; the precision of the pumps is limited when the lower flow rate accuracy boundary is exceeded. The residence time distributions obtained for the PDMAm tracer are broader than the DMAm equivalent; viscosity has been shown by Reis *et al.*[9] to effect the residence time distributions

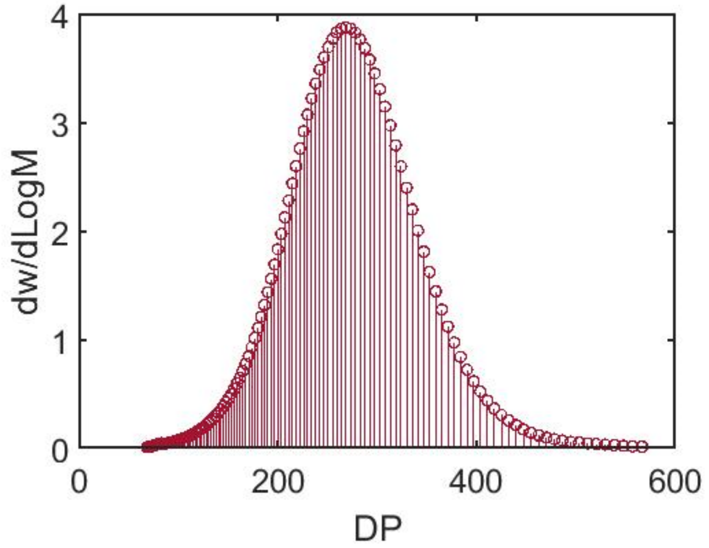

Figure S7. Distribution of molecular weights as obtained by GPC for PDMAm$_{200}$ at 98 % conversion.

Here, it is assumed that each degree of polymerization eluted in the GPC experiences its own RTD due to laminar flow. Firstly, each chain in the MWD from batch assuming ideal mixing is multiplied through by the residence time distribution function.

$$E(MW) = E(\theta).\,dw/dlogM \qquad [S\ 62]$$

$$MW = \theta.\,M \qquad [S\ 63]$$

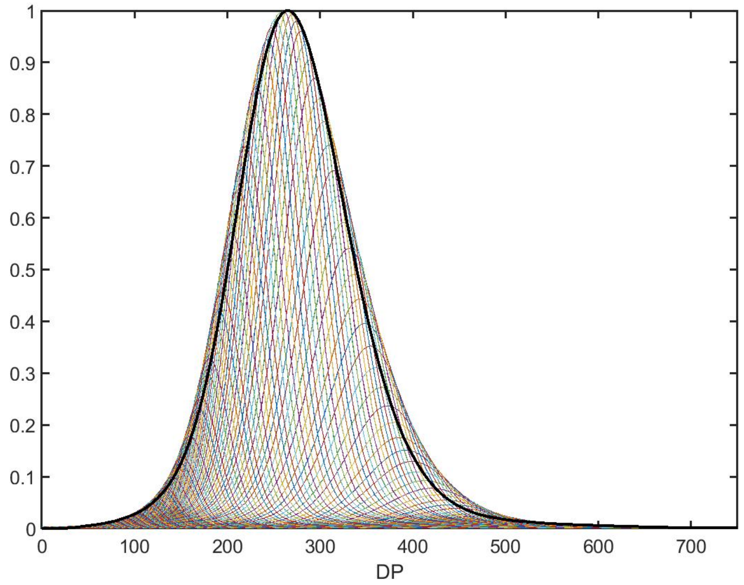

Figure S8. RTD (coloured line super Imposed onto each of the stems seen in the MW with the batch molecular weight distribution overlayed

A Gaussian fitting function (*gmfitdist* MATLAB) is used to fit coefficients, μ, σ *and* c.

$$G(x) = \frac{1}{\sigma\sqrt{2\pi}} e^{-\frac{1}{2}\left(\frac{x-\mu}{\sigma}\right)^2} + c \qquad [S\ 64]$$

The individual Gaussians are then simulated on a known linearly spaced X scale between a DP 0 and 900 and convoluted to give Figure S9

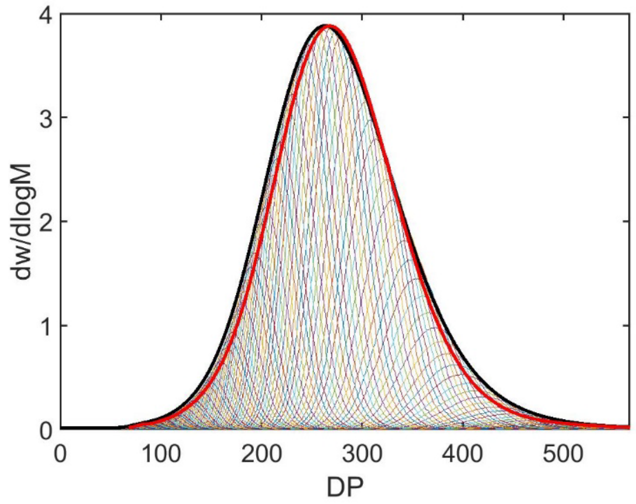

Figure S9. Comparison of the original MWD (Red) to the simulated MWD accounting for RTD effects

The *Đ* [S 67] of the original MWD and the new MWD were then calculated using the ratio of the number average, $\boldsymbol{M_n}$ [S 65], $\boldsymbol{M_w}$ [S 66]. The *Đ* of the batch was then subtracted from the *Đ* of the new batch to give the contribution . Table S1

$$\overline{M_n} = \frac{\sum N_i M_i}{\sum N_i} \qquad [S\ 65]$$

$$\overline{M_w} = \frac{\sum N_i M_i^2}{\sum N_i M_i} \qquad [S\ 66]$$

$$Đ = \frac{\overline{M_w}}{\overline{M_n}} \qquad [S\ 67]$$

Table S1. Simulated RTD Contribution calculations compared to GPC chromatograms from flow

	Experimental[GPC]	With RTD	Contribution
Batch	1.0725		
Flow	1.1351	1.1022	0.0277

5. Experimental

The flow reactor platform

A reactor comprising of three computer controlled JASCO-PU 980 and 1580, 2 packed bed mixers (see below), an aluminium heating block fitted with 2 Elmatic Max K cartridge heaters regulated by a Eurotherm 3210 temperature controller. 1/16" stainless steel tubing with an internal volume of 5 mL was coiled around the heating block forming the reactor vessel. The Equipment was controlled by a bespoke interface in MATLAB and kinetic experiments were performed autonomously. GPC data was collected off-line for accuracy using an Agilent Infinity 1260 fitted with a guard column and two 5 μm mixed-C columns with RI detection and UV/Vis at 309 nm with DMF treated with LiBr 0.1 w/w % as the eluent. The GPC system was calibrated with poly(methyl methacrylate) calibrants.A Magritek SpinSolve ultra 60 was used for monitoring of conversion using a presaturation solvent suppression method (1s saturation at 3.3 ppm of -65 dB, 7 μs excitation pulse, with an acquisition time of 6.4 s, a repetition time of 10 s for 2 scans. Kinetic samples were taken after 3 reactor volumes to ensure steady state had been reached.

NMR conversion was found using

$$\alpha \;= 1 - \frac{2z}{(y+\frac{x}{2})}$$

DMAm:TTC1:VA044 in water

N,N-dimethyl acrylamide (60 g, 100 eq) and 3-((((1-carboxyethyl)thio)carbonothioyl)thio)propanoic acid (1.54 g, 1 eq) were dissolved in deionised water (67.6 g). To a separate flask 2,2'-Azobis[2-(2-imidazolin-2-yl)propane]dihydrochloride (0.49 g, 0.1 eq) was dissolved in 102.9 g of deionised water. A third flask was dosed with deionised water only. The 3 vessels were degassed under an atmosphere of nitrogen for 20 mins prior to reaction then kept under nitrogen for the rest of the experimental time. Off-line NMR and offline GPC was used in this case.

For the batch experiments a Deep Matter digital glassware probe was used. A 3 necked RBF was dosed with DMAm (15.0 g, 0.15 mol, 100 eq), 3-((((1-carboxyethyl)thio)carbonothioyl)thio)propanoic acid (0.385 g, 1.51 mmol, 1 eq) and 2,2'-Azobis[2-(2-imidazolin-2-yl)propane]dihydrochloride (9.7 mg, 0.030 mmol, 0.02 eq) was dissolved in water (35.9 g) . The solution was degassed under nitrogen for 20 mins and submerged into a preheated oil bath set to 80 °C. Samples were analysed by offline NMR and offline GPC.

DMAm:TTC2:VA044 in water

N,N-dimethyl acrylamide (60 g, 200 eq) and 2-(Butylthiocarbonothioylthio)propanoic acid (1.72 g, 1 eq)was dissolved in deionised water (41.2 g). To a separate flask 2,2'-Azobis[2-(2-imidazolin-2-yl)propane]dihydrochloride (0.49 g, 0.1 eq) was dissolved in 102.9 g of deionised water. A third flask was dosed with deionised water only. The 3 vessels were degassed under an atmosphere of nitrogen for 20

mins prior to reaction then kept under nitrogen for the rest of the experimental time. The reagents were then diluted in stream to 30 w/w %

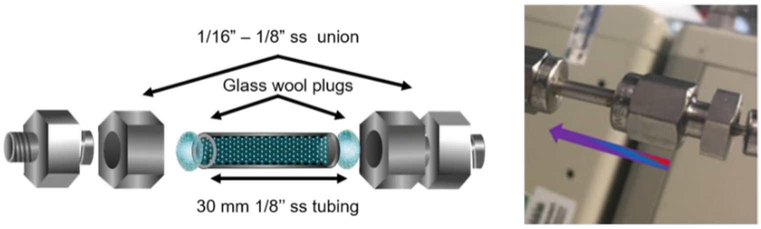

Figure S10. Packed bed mixer used for additional mixing of the streams used in this paper.

DMAm (dimethylacrylamide, 99% purity purchased from Sigma Aldrich, CAS -2680-03-7) purchased from Sigma Aldrich, TT1(3-((((1-carboxyethyl)thio)carbonothioyl)thio)propanoic acid, 95% purity, CAS - 870451-09-5), TT2(2-(Butylthiocarbonothioylthio)propanoic acid, 95 % purity, CAS 480436-46-2) both purchased Boron Molecular, ACVA (4,4′-Azobis(4-cyanovaleric acid) purchased from Sigma Aldrich, 98 % purity, CAS 2638-94-0), VA044 (2,2'-Azobis[2-(2-imidazolin-2-yl)propane]dihydrochloride, CAS 27776-21-2) purchased from Wako Fujifilm and deionised water was used in this paper.

6. **Rate Constants**

Table S2. Arrhenius and rate parameters used in the simulation of DMAm : TTC1 : VA044 in water 30 w/w % at 80 (100:1:0.02)

	E_a		A		$k(80°C)$		Ref.
k_p	14.1	$kJ\ mol^{-1}$	1.10×10^7	$M^{-1}s^{-1}$	9.03×10^4	$M^{-1}s^{-1}$	10
$k_t = k_{td} = k_{tc}$ *	25.0	$kJ\ mol^{-1}$	2.00×10^{11}	$M^{-1}s^{-1}$	4.00×10^7	$M^{-1}s^{-1}$	11
k_a*	0	$kJ\ mol^{-1}$	6.00×10^6	$M^{-1}s^{-1}$	6.00×10^6	$M^{-1}s^{-1}$	12
k_β*					2.00×10^4	s^{-1}	13
k_d	108.2	$kJ\ mol^{-1}$	1.28×10^{12}	s^{-1}	5.48×10^{-4}	s^{-1}	14
Other parameters							
f	0.50			α_s *	0.40	11	
k_{ct}	0.25 k_t			α_l*	0.18	11	
K*	300			L_c	50	11	

Table S3. Arrhenius and rate parameters used in the simulation of DMAm : TTC2 : ACVA in water 30 w/w (200:1:0.02)

	E_a		A		$k(85°C)/(90°C)$		Ref.
k_p	14.1	$kJ\ mol^{-1}$	1.10×10^{7}	$M^{-1}s^{-1}$	9.66×10^{4}<br>1.03×10^{5}	$M^{-1}s^{-1}$	10
$k_t = k_{td} = k_{tc}$ *	25.0	$kJ\ mol^{-1}$	$2.00\ \times 10^{11}$	$M^{-1}s^{-1}$	4.52×10^{7}<br>5.07×10^{7}	$M^{-1}s^{-1}$	11
k_a*	0	$kJ\ mol^{-1}$	5.50×10^{6}	$M^{-1}s^{-1}$	7.00×10^{6}	$M^{-1}s^{-1}$	15
k_β*					7.00×10^{4}	s^{-1}	15
k_d	132.4	$kJ\ mol^{-1}$	1.37×10^{15}	s^{-1}	6.69×10^{-5}<br>1.23×10^{-4}	s^{-1}	14
Other parameters							
f	0.70		α_s*	0.40	11		
k_{ct}	0.25 k_t		α_l*	0.18	11		
K*	100		L_c*	50			

1

Table S4. Arrhenius and rate parameters used in the simulation of AAm : TTC3 : VA044 in water 15 w/w % at 45 (300:1:0.1).

		E_a	A			$k(45°C)$	Ref.
k_p	18.1	$kJ\ mol^{-1}$	1.47×10^{8}	$M^{-1}s^{-1}$	1.27×10^{5}	$M^{-1}s^{-1}$	16
$k_t = k_{td} = k_{tc}$ *	19.1	$kJ\ mol^{-1}$	5.30×10^{11}	$M^{-1}s^{-1}$	3.88×10^{8}	$M^{-1}s^{-1}$	11
k_a*	0	$kJ\ mol^{-1}$	5.00×10^{7}	$M^{-1}s^{-1}$	7.00×10^{7}	$M^{-1}s^{-1}$	17
k_β*					1.67×10^{2}	s^{-1}	15
k_d	108.2	$kJ\ mol^{-1}$	5.53×10^{12}	s^{-1}	2.19×10^{-5}	s^{-1}	14
Other parameters							
f	0.55		α_s*	0.50	11		
k_{ct}	0.25 k_t		α_l*	0.15	11		
K	25000		L_c*	30	11		

Table S5. Arrhenius and rate parameters used in the simulation of AA:TTC4:ACVA in water 13 w/w % at 69(100:1:0.1)

	E_a		A			$k(69\ °C)$	Ref.
k_p	15.0	$kJ\ mol^{-1}$	6.60×10^{7}	$M^{-1}s^{-1}$	$3.89\ \times 10^{5}$	$M^{-1}s^{-1}$	16
$k_t = k_{td} = k_{tc}$ *	15.0	$kJ\ mol^{-1}$	1.60×10^{12}	$M^{-1}s^{-1}$	8.20×10^{9}	$M^{-1}s^{-1}$	11
k_a*	0	$kJ\ mol^{-1}$	2.00×10^{7}	$M^{-1}s^{-1}$	2.00×10^{7}	$M^{-1}s^{-1}$	17
k_β*					2.00×10^{2}	s^{-1}	15
k_d	132.8	$kJ\ mol^{-1}$	3.62×10^{15}	s^{-1}	1.93×10^{-5}	s^{-1}	14
Other parameters							
f	0.70		α_s*	0.80	11		
k_{ct}	0.25 k_t		α_l*	0.16	11		
K*	3000		L_c*	30	11		

Table S6. Arrhenius and rate parameters used in the simulation of MA:TTC5:AIBN in water 30 w/w % at 50 (100:1:0.1)

	E_a		A			$k(50\ °C)$	Ref.
k_p	17.4	$kJ\ mol^{-1}$	1.87×10^7	$M^{-1}s^{-1}$	2.82×10^5	$M^{-1}s^{-1}$	18
$k_t = k_{td} = k_{tc}$ *	10.0	$kJ\ mol^{-1}$	2.30×10^{10}	$M^{-1}s^{-1}$	5.56×10^8	$M^{-1}s^{-1}$	19
k_a*	0	$kJ\ mol^{-1}$	2.00×10^6	$M^{-1}s^{-1}$	7.00×10^6	$M^{-1}s^{-1}$	17
k_β*					2.00×10^3	s^{-1}	15
k_d	132.8	$kJ\ mol^{-1}$	2.37×10^{15}	s^{-1}	2.16×10^{-6}	s^{-1}	14
Other parameters							
f	0.70		α_s*	0.74	11		
k_{ct}	0.25 k_t		α_l*	0.15	11		
K*	1000		L_c*	30	11		

*Approximate values based on fitting with close literature values

References

(1) Wang, A. R.; Zhu, S. Calculations of Monomer Conversion and Radical Concentration in Reversible Addition-Fragmentation Chain Transfer Radical Polymerization. *Macromol. Theory Simulations* **2003**, *12* (9), 663–668. https://doi.org/10.1002/mats.200350025.

(2) Barner-kowollik, C.; Buback, M.; Charleux, B.; Coote, M. L.; Drache, M.; Fukuda, T.; Goto, A.; Klumperman, B.; Lowe, A. B.; Mcleary, J. B.; et al. Mechanism and Kinetics of Dithiobenzoate-Mediated RAFT Polymerization . I . The Current Situation. **2006**, *44*, 5809–5831. https://doi.org/10.1002/pola.

(3) Barner-Kowollik, C.; Quinn, J. F.; Morsley, D. R.; Davis, T. P. Modeling the Reversible Addition-Fragmentation Chain Transfer Process in Cumyl Dithiobenzoate-Mediated Styrene Homopolymerizations: Assessing Rate Coefficients for the Addition-Fragmentation Equilibrium. *J. Polym. Sci. Part A Polym. Chem.* **2001**, *39* (9), 1353–1365. https://doi.org/10.1002/pola.1112.

(4) Moad, G.; Barner-Kowollik, C. The Mechanism and Kinetics of the RAFT Process: Overview, Rates, Stabilities, Side Reactions, Product Spectrum and Outstanding Challenges. In *Handbook of RAFT Polymerization*; 2008. https://doi.org/10.1002/9783527622757.ch3.

(5) Mastan, E.; Li, X.; Zhu, S. Modeling and Theoretical Development in Controlled Radical Polymerization. *Prog. Polym. Sci.* **2015**, *45*, 71–101. https://doi.org/10.1016/j.progpolymsci.2014.12.003.

(6) Perrier, S. 50th Anniversary Perspective: RAFT Polymerization - A User Guide. *Macromolecules*. 2017, pp 7433–7447. https://doi.org/10.1021/acs.macromol.7b00767.

(7) Mastan, E.; Zhu, S. A Molecular Weight Distribution Polydispersity Equation for the ATRP System: Quantifying the Effect of Radical Termination. *Macromolecules* **2015**, *48* (18), 6440–6449. https://doi.org/10.1021/acs.macromol.5b01525.

(8) Wang, T. T.; Wu, Y. Y.; Luo, Z. H.; Zhou, Y. N. "living" Polymer Dispersity Quantification for Nitroxide-Mediated Polymerization Systems by Mimicking a Monodispersed Polymer Blending Strategy. *Macromolecules* **2020**, *53* (24), 10813–10822. https://doi.org/10.1021/acs.macromol.0c02029.

(9) Reis, M. H.; Varner, T. P.; Leibfarth, F. A. The Influence of Residence Time Distribution on Continuous-Flow Polymerization. *Macromolecules* **2019**, *52* (9), 3551–3557. https://doi.org/10.1021/acs.macromol.9b00454.

(10) Schrooten, J.; Lacík, I.; Stach, M.; Hesse, P.; Buback, M. Propagation Kinetics of the Radical Polymerization of Methylated Acrylamides in Aqueous Solution. *Macromol. Chem. Phys.* **2013**, *214* (20), 2283–2294. https://doi.org/10.1002/macp.201300357.

(11) Kattner, H.; Buback, M. Termination and Transfer Kinetics of Acrylamide Homopolymerization in Aqueous Solution. *Macromolecules* **2015**, *48* (20).

https://doi.org/10.1021/acs.macromol.5b01921.

(12) Vandenbergh, J.; De Moraes Ogawa, T.; Junkers, T. Precision Synthesis of Acrylate Multiblock Copolymers from Consecutive Microreactor RAFT Polymerizations. *J. Polym. Sci. Part A Polym. Chem.* **2013**, *51* (11), 2366–2374. https://doi.org/10.1002/pola.26593.

(13) Meiser, W.; Barth, J.; Buback, M.; Kattner, H.; Vana, P. EPR Measurement of Fragmentation Kinetics in Dithiobenzoate-Mediated RAFT Polymerization. *Macromolecules* **2011**, *44* (8). https://doi.org/10.1021/ma102491x.

(14) Wako. Azo Polymerization Initiators Comprehensive Catalog. *Wako Cat.* **2016**.

(15) Alberti, A.; Benaglia, M.; Fischer, H.; Guerra, M.; Laus, M.; Macciantelli, D.; Postma, A.; Sparnacci, K. An ESR Approach to the Estimation of the Rate Constants of the Addition and Fragmentation Processes Involved in the RAFT Polymerization of Styrene. *Helv. Chim. Acta* **2006**, *89* (10). https://doi.org/10.1002/hlca.200690200.

(16) Lacík, I.; Chovancová, A.; Uhelská, L.; Preusser, C.; Hutchinson, R. A.; Buback, M. PLP-SEC Studies into the Propagation Rate Coefficient of Acrylamide Radical Polymerization in Aqueous Solution. *Macromolecules* **2016**, *49* (9), 3244–3253. https://doi.org/10.1021/acs.macromol.6b00526.

(17) Lin, C. Y.; Coote, M. L. How Well Can Theory Predict Addition-Fragmentation Equilibrium Constants in RAFT Polymerization? *Aust. J. Chem.* **2009**, *62* (11). https://doi.org/10.1071/CH09269.

(18) Haehnel, A. P.; Wenn, B.; Kockler, K.; Bantle, T.; Misske, A. M.; Fleischhaker, F.; Junkers, T.; Barner-Kowollik, C. Solvent Effects on Acrylate Kpin Organic Media? - A Systematic PLP-SEC Study. *Macromol. Rapid Commun.* **2014**, *35* (23), 2029–2037. https://doi.org/10.1002/marc.201400479.

(19) Kattner, H.; Buback, M. Termination, Propagation, and Transfer Kinetics of Midchain Radicals in Methyl Acrylate and Dodecyl Acrylate Homopolymerization. *Macromolecules* **2018**, *51* (1). https://doi.org/10.1021/acs.macromol.7b02241.
